# Acid catalyzed one-pot approach towards the synthesis of curcuminoid systems: unsymmetrical diarylidene cycloalkanones, exploration of their single crystals, optical and nonlinear optical properties†

**DOI:** 10.1039/d2ra07681k

**Published:** 2023-02-02

**Authors:** Akbar Ali, Zia Ud Din, Muhammad Ibrahim, Muhammad Ashfaq, Shabbir Muhammad, Dania Gull, Muhammad Nawaz Tahir, Edson Rodrigues-Filho, Abdullah G. Al-Sehemi, Muhammad Suleman

**Affiliations:** a Department of Chemistry, Government College University Faisalabad, 38000-Faisalabad Pakistan; b LaBioMMi, Departamento de Química, Universidade Federal de São Carlos CP 676, São Carlos SP 13.565-905 Brazil; c Department of Applied Chemistry, Government College University Faisalabad Pakistan ibrahimchem@gmail.com; d Department of Physics, University of Sargodha Sargodha Pakistan ashfaq.muhammad@uos.edu.pk muhammadashfaq1400@gmail.com; e Department of Chemistry, College of Science, King Khalid University P.O. Box 9004 Abha 61413 Saudi Arabia; f Department of Chemistry, Riphah International University Faisalabad Campus Pakistan

## Abstract

In the present study crystalline unsymmetrical diarylidene ketone derivatives BNTP and BDBC have been prepared by two sequential acid catalyzed aldol condensation reactions in a one pot manner. The crystal structures of both compounds were confirmed by single crystal X-ray diffraction analysis which revealed the presence of H-bonding interactions of type C–H⋯O, along with weak C–H⋯π and weak π⋯π stacking interactions that are involved in the crystal stabilization of both organic compounds. Hirshfeld surface analysis is carried out for the broad investigation of the intermolecular interactions in both compounds. The quantum chemical investigation was performed on the optimized molecular geometries of BNTP and BDBC to calculate optical and nonlinear optical (NLO) properties. The density functional theory (DFT) study showed that the third-order NLO polarizabilities of compounds BNTP and BDBC are found to be 226.45 × 10^−36^ esu and 238.72 × 10^−36^ esu, respectively, which indicates noticeable good NLO response properties. Additionally, the BNTP and BDBC molecules also showed the HOMO–LUMO orbital gaps of 5.96 eV and 6.06 eV, respectively. Furthermore, the computation of UV-visible spectra of the titled compounds indicated a limited and/or no absorption above the 400 nm region, directing a good transparency and NLO property trade-off for both synthesized compounds that may play a significant contribution in the future for optoelectronic technologies.

## Abstract 2

In the present study crystalline unsymmetrical diarylidene ketone derivatives BNTP and BDBC have been prepared by two sequential acid catalyzed aldol condensation reactions in a one pot manner.

## Graphical abstract

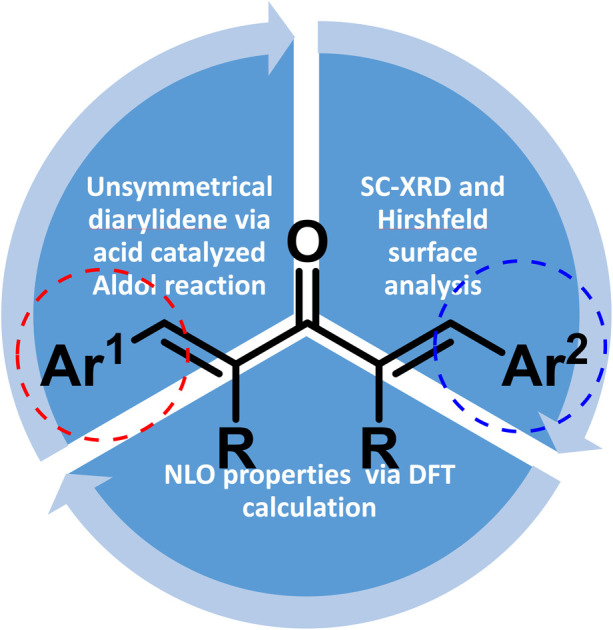

## Introduction

The increasing global population and climate change has a meaningful effect on social health. The increase in pollution, greenhouse gases, deforestation, global warming, floods *etc.* have affected the entire world. Moreover, human health is also facing risks on account of the frequent use and misuse of anti-infectious medication causing the emergence of multidrug resistant species. Thus, constant efforts are required to develop new therapeutic drugs with effective potential against the resistant microbes. In this regard, curcumin (derived from rhizome) is considered to be one of most important classes of natural products with numerous biological and chemical applications.^1^ Traditionally, curcuminoids (naturally found in the perennial herb curcuma/turmeric of the ginger family, Zingiberaceae) have been used as herbal supplements and as spices in the south Asia reign. Beside this, the powdered form of turmeric has find vast applications in medicines,^2^ cosmetics, food flavoring, and fabric dying *etc.*^3^ Symmetrical and unsymmetrical curcuminoids are pharmaceutically active compounds,^4^ exhibiting various medicinal uses such as anti-parasitic,^5^ antibacterial,^6^ antioxidant,^7^ antimitotic,^8^ antimalarial,^9^ antitumor,^10^ neuro-protective,^11^ antimicrobial,^12^ antifungal,^13^ anti-inflammatory,^14^ anti-tubercular^14^ anti-fertility,^15^ anti-viral,^16^ anti-tubulin^17^ and anticancer.^18^

Furthermore, Castro *et al.*,^19^ reported the conjugates of fullerene-curcumin for biological and photovoltaic applications and the reported conjugates have displayed HOMO/LUMO energy levels comparable to perovskite solar cells. Curcuminoid derivatives as borondifluoride complexes has also reported for optical and photovoltaic behavior as donor–acceptor–donor motifs.^20^ Saeed *et al.*,^21^ have reported the nonlinear optical properties of dihydropyridone curcumin derivatives through determination of nonlinear refractive index. The study of NLO aspects has captivated the interest of the scientific community (experimental and theoretical researchers) as materials with appreciable NLO capabilities have found numerous applications in the scientific arena,^22^ especially in the fields of biophysics, medicine, solid physics, atomic science, material science and chemical dynamics are worth mentioning.^23^ It is important to reference that organic materials have found considerable appreciation in the scientific community because of strong NLO capabilities accompanied with substantial advantages over the inorganic counterparts due to their low toxic nature, little cost, and simplicity in their synthetic process.^24^ Density functional theory (a computational modelling method) is commonly applied as an effective tool to analyze the NLO characteristics of organic materials.

**Scheme 1:**
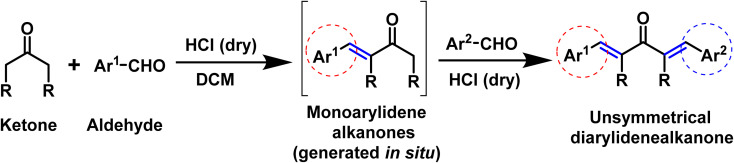
Acid catalyzed synthesis of unsymmetrical diarylidene alkanones compounds.

In this scenario, here we are presenting our findings regarding the acid catalyzed one-pot synthesis of unsymmetrical diarylidene cycloalkanones and its single crystal exploration accompanied with DFT exploration (Scheme 1).

## Experimental section

### Chemistry

Standard chemicals were purchased and used without further purification from the reputed chemical suppliers like Sigma-Aldrich, Acros Chemicals, Macklin, and TCI. The reaction progress was observed by using TLC plates (pre-coated silica gel G-25-UV254). The dry HCl used as catalyst was generated by the reaction of sodium chloride and concentrated sulfuric acid and passed through reaction mixture *via* glass delivery tube.

## General procedure

### Preparation of compound BNTP

**Scheme 2:**
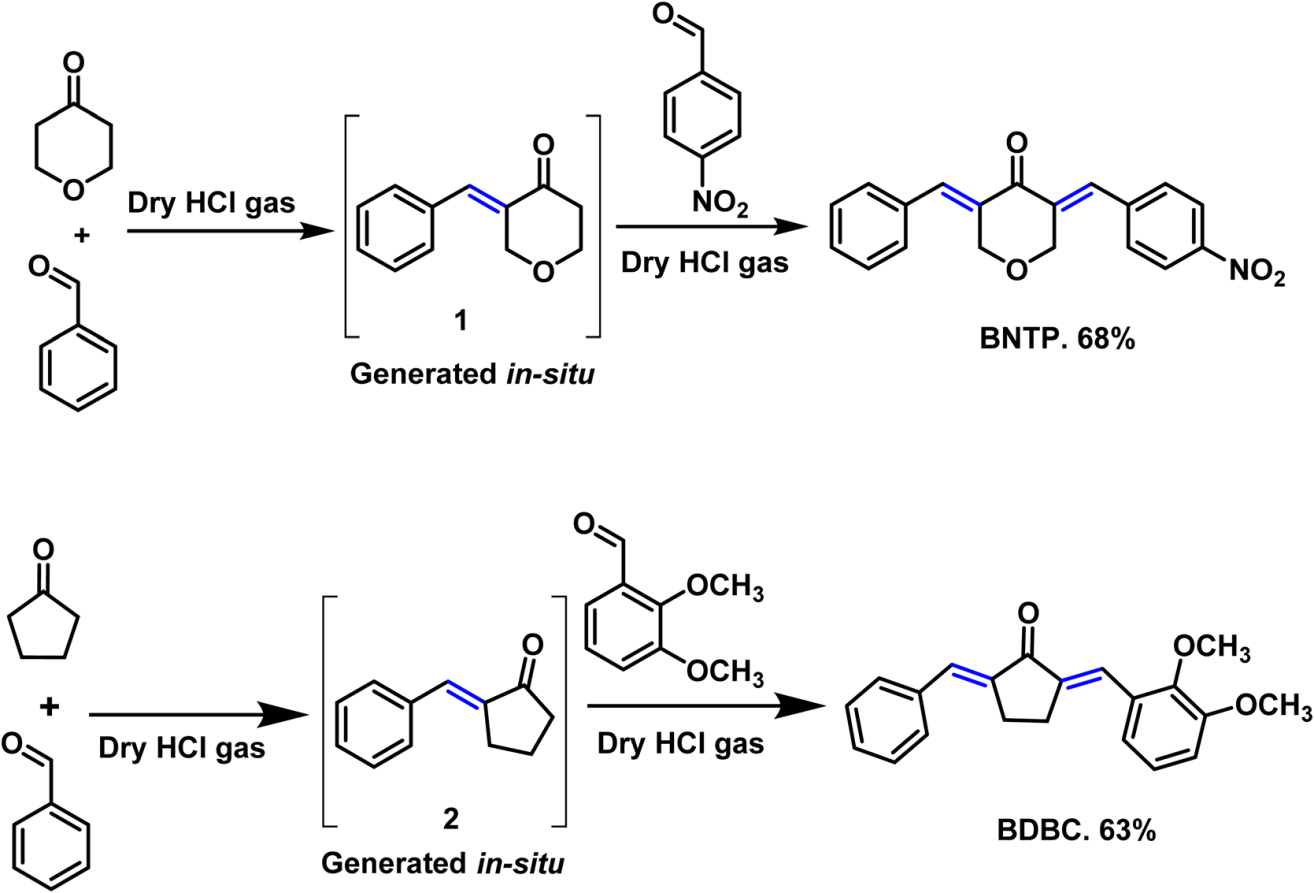
Synthetic route for the accomplishment of the organic crystalline compounds: BNTP and BDBC.

Tetrahydro-4*H*-pyran-4-one and benzaldehyde were taken (1 : 1) in a two necked round bottom flask (50 mL) using chloroform as solvent (20 mL). Dry HCl gas was passed through this reaction mixture till its color turns into red. Stirring was sustained for 4 to 6 hours. After accomplishment of intermediate 1*in situ* (monitered by TLC), 1 equivalent of 4-nitrobenzaldehyde was added and the reaction mixture was further stirred for 5 hours.^25,26^ After completion (TLC indication), the solvent of the reaction mixture was evaporated by rotary evaporator and the residue was diluted using ethyl acetate. The extraction was performed with NaHSO_3_ solution and the organic layer was dried with anhydrous Na_2_SO_4_. The crude product was purified using column chromatography. Finally, the recrystallization in ethanol gave the titled compound BNTP in 68% yield (Scheme 2).

### Preparation of compound BDBC

Cyclopentanone and benzaldehyde were taken (1 : 1) in a two necked round bottom flask (50 mL). Dry HCl gas was passed through this reaction mixture till its color turns into red. Stirring was sustained for 4 to 6 hours.^25,26^ After accomplishment of intermediate 2 *in situ* (monitered by TLC), 1 equivalent of 2,3-dimethoxy benzaldehyde was added and the reaction mixture was further stirred for 5 hours. After completion (TLC indication), the solvent of the reaction mixture was evaporated by rotary evaporator and the residue was diluted using ethyl acetate. The extraction was performed with NaHSO_3_ solution and the organic layer was dried with anhydrous Na_2_SO_4_. The crude product was purified using column chromatography. Finally, the recrystallization in ethanol gave the titled compound BDBC in 63% yield (Scheme 2).

### Characterization

The unsymmetrical diarylidene ketone derivatives, BNTP and BDBC have been prepared by the two sequential acid catalyzed aldol condensation reactions in a one pot manner. The single crystal XRD data of both compounds was collected on Bruker Kappa Apex-II CCD diffractometer with molybdenum x-rays source. The SHELXT-2014^27^ and SHELXL 2019/2^28^ software were used for structure solution and refinement, respectively. ORTEP-III,^29^ PLATON^30^ and Mercury version 4.0^31^ were used for the graphical representations of single crystal XRD results. All the non-hydrogen atoms were refined by using anisotropic displacement parameters. The isotropic displacement parameters were assigned to H-atoms. The hydrogen atoms were refined by using riding model. The details of SC-XRD analysis are given in Table 1. The ^1^H and ^13^C NMR spectra of BNTP and BDBC are shown in Fig. S1 and S2,† respectively. The NMR data is given below while these crystalline compounds were also confirmed by the single crystal analysis.

**Table 1:** Experimental details of BNTP–BDBC

Crystal data	BNTP	BDBC
CCDC	2172047	2172048
Chemical formula	C_19_H_15_NO_4_	C_21_H_20_O_3_
*M*_r_	321.32	320.37
Crystal system, space group	Monoclinic, *P*2_1_/*n*	Orthorhombic, *Pbca*
Temperature (*K*)	296	150
*a*, *b*, *c* (Å)	7.5877 (4), 7.2192 (4), 28.0023 (14)	16.154 (2), 6.9813 (10), 28.997 (4)
*α*, *β*, *γ*	90, 90.507 (1), 90	90, 90, 90
V (Å^3^)	1533.83 (14)	3270.2 (8)
*Z*	4	8
Density (calculated) g cm^−3^	1.391	1.301
F(000)	672	1360
Radiation type	Mo *K*α	Mo Kα
Wavelength (*λ*)	0.71073 Å	0.71073 Å
*μ* (mm^−1^)	0.098	0.086
Crystal size (mm)	0.38 × 0.38 × 0.19	0.36 × 0.35 × 0.19

**Data collection**		
Diffractometer	Bruker APEXII CCD diffractometer	Bruker APEXII CCD diffractometer
Absorption correction	Multi-scan (SADABS; Bruker, 2007)	Multi-scan (SADABS; Bruker, 2007)
No. of measured, independent and observed [*I* > 2*σ*(*I*)] reflections	24896, 3262, 2850	57033, 2992, 2623
*R*_int_	0.019	0.026
Theta range for data collection (°)	1.454 to 26.729	1.404 to 25.347
Index ranges	−9≤ *h* ≤ 9, −9≤ *k* ≤ 9, −35 ≤ *l* ≤ 35	−19 ≤ *h* ≤ 19, −8≤ *k* ≤ 5, −34 ≤ *l* ≤ 34
(sin *θ*/*λ*)_max_(Å^−1^)	0.633	0.602

**Data refinement**		
*R*[*F*^2^ > 2σ(*F*^2^)], *wR*(*F*^2^), *S*	0.041, 0.110, 1.03	0.055, 0.116, 1.16
No. of reflections	3262	2992
No. of parameters	217	219
H-atom treatment	H-atom parameters constrained	H-atom parameters constrained
Δ*ρ*_max_, Δ*ρ*_min_(*e* Å^−3^)	0.19, −0.19	0.22, −0.17

#### 3-((*E*)-benzylidene)-5-((*E*)-4-nitrobenzylidene)tetrahydro-4*H*-pyran-4-one (BNTP)

^1^H NMR (400 MHz, CDCl_3_) *δ* 8.34–8.27 (m, 2H), 7.87 (dt, *J* = 7.7, 1.9 Hz, 2H), 7.51–7.43 (m, 5H), 7.36 (dd, *J* = 7.7, 1.6 Hz, 2H), 4.98 (d, *J* = 1.9 Hz, 2H), 4.93 (d, *J* = 2.0 Hz, 2H). ^13^C NMR (101 MHz, CDCl_3_) *δ* 185.0, 147.7, 141.0, 137.6, 136.2, 134.4, 133.2, 132.5, 130.8, 130.6, 129.8, 128.8, 123.9, 68.7, 68.3. FT-IR (cm^−1^): ν̃ 3053 (=C–H), 2833, 2924 (=C–H for anti-symmetry, symmetry), 1670 (C=O), 1612 (C=C), 1492 (–CH_2_), 1444 (–NO_2_) 1265 (C–O).

#### 2-((*E*)-benzylidene)-5-((*E*)-2,3-dimethoxybenzylidene)cyclopentan-1-one (BDBC)

^1^H NMR (400 MHz, CDCl_3_) *δ* 7.96 (t, *J* = 2.4 Hz, 1H), 7.60 (d, *J* = 6.9 Hz, 3H), 7.47–7.41 (m, 2H), 7.41–7.35 (m, 1H), 7.18 (dd, *J* = 7.9, 1.4 Hz, 1H), 7.11 (t, *J* = 8.0 Hz, 1H), 6.96 (dd, *J* = 8.1, 1.4 Hz, 1H), 3.92–3.87 (m, 6H), 3.10–3.02 (m, 4H). ^13^C NMR (100 MHz, CDCl_3_) *δ* 196.3, 153.0, 149.3, 138.4, 137.5, 136.3, 135.9, 133.7, 130.7, 130.1, 129.3, 128.8, 128.3, 123.8, 121.6, 113.6, 61.5, 55.9, 26.6. FT-IR (cm-1): ν̃ 3053 (=C–H), 2833, 2924 (=C–H for anti-symmetry, symmetry), 1670 (C=O), 1612 (C=C), 1492 (–CH_2_), 1265 (C–O).

**Fig. 1:**
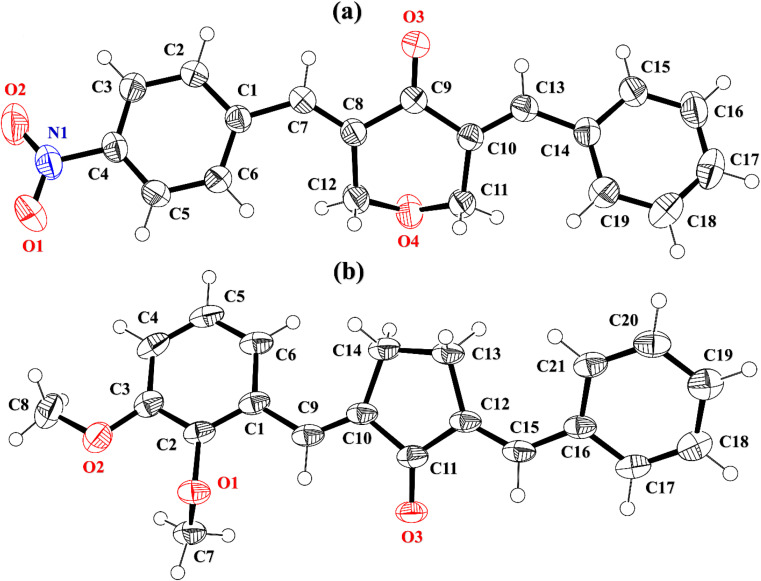
ORTEP diagram of BNTP, BDBC that are drawn at probability level of 50%. H-atoms are shown by small circles of arbitrary radii.

**Fig. 2:**
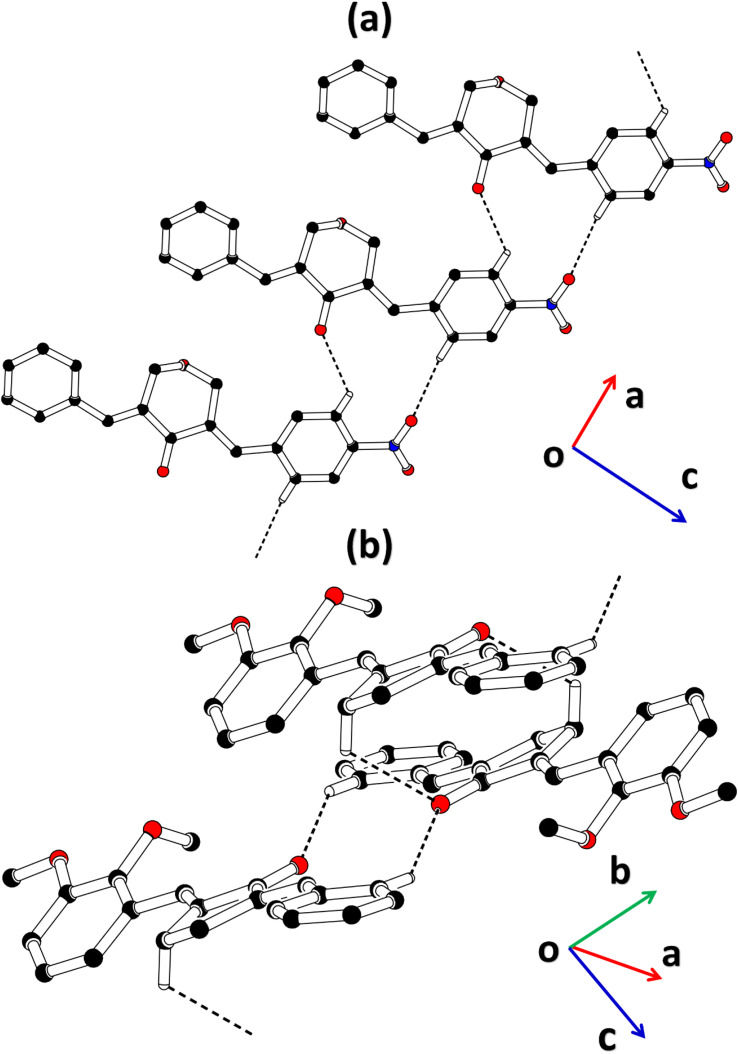
Packing diagram of (a) BNTP, (b) BDBC. Selected H-atoms are shown for clarity.

**Fig. 3:**
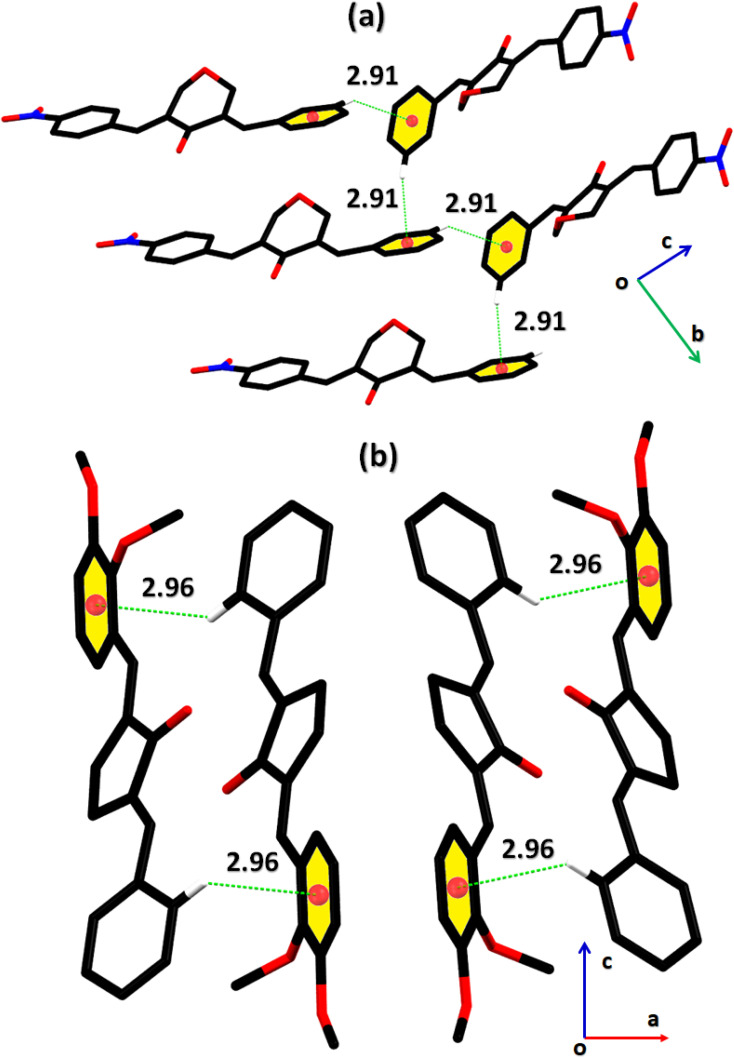
Graphical representation of C–H⋯π in (a) BNTP, (b) BDBC. Selected H-atoms are shown for clarity.

The cambridge structure data base search conformed that the crystal structures of BNTP and BDBC are novel. In BNTP (Fig. 1a and Table 1), the first benzyl group A (C1–C7) and the second benzyl group B (C13–C19) are planar with root mean square (r.m.s.) deviation of 0.0123 and 0.0160 Å, respectively with the dihedral A/B of 7.4 (6)°. The nitro group C makes the dihedral of 13.7 (1)° with the mean plane of its present group A. The tetrahydro-4*H*-pyran-4-one ring D (C8–C12/O3/O4) is puckered with puckering parameters (*Q* = 0.5328 (15) Å, *θ* = 59.51 (16)°, *φ* = 2.9 (2)°) and adopts a conformation very similar to envelope conformation. The dihedral angles D/A and D/B are 25.4 (4)° and 32.2 (4)°, respectively. The molecules of BNTP are interlinked in the form of dimers through C–H⋯O bonding to complete R^2^_2_(12) loop, one of the O-atom of nitro group acts as H-bond acceptor for meta-positioned CH of phenyl ring attached with nitro group (Fig. 2a). Carbonyl O-atom acts as H-bond acceptor for ortho-positioned CH of phenyl ring attached with nitro group. C8 zigzag chain is formed by C5–H5⋯O3 bonding whereas C6 zigzag chain is formed by C5–H5⋯O3 bonding. Both chains run along a-axis. The crystal packing is further stabilized by weak C–H⋯π interaction that interlinked in the molecules in the form of infinite chain along *b*-axis with H⋯π distance of 2.92 Å (Table 2, Fig. 3a). Off-set π⋯π stacking interaction also found in the crystal packing with inter-centroid separation 3.8474(9) to 5.4822(9) Å.

**Table 2:** Hydrogen-bond geometry (Å, °) and C–H⋯π interaction for BNTP and BDBC^a^

BNTP	D–H⋯A	D–H	H⋯A	D⋯A	<(D–H⋯A)
	C5–H5⋯O3^i^	0.93	2.56	3.1620 (18)	123
	C2–H2⋯O1^ii^	0.93	2.59	3.4102 (19)	148
	C–H⋯π	C–H	H⋯π	C⋯π	<(C–H⋯π)°
	C18–H18⋯Cg1^iii^	0.93	2.91	3.6811 (17)	141
BDBC	D—H···A	D—H	H⋯A	D⋯A	<(D—H⋯A)°
	C14–H14B⋯O3^iv^	0.99	2.60	3.206 (3)	120
	C17–H17⋯O3^v^	0.95	2.49	3.319 (3)	146
	C–H⋯π	C–H	H⋯π	C⋯π	<(C–H⋯π)°
	C17–H17⋯Cg1^iv^	0.95	2.96	3.490 (3)	116

a Symmetry codes: (i) *x* − 1, *y*,*z*; (ii) *x* + 1, *y*, *z*; (iii) *x* − 3/2, *y* + 1/2, −*z* + 1/2; (iv) −*x*, −*y*, −*z*; (v) −*x*, −*y*−1, −*z*.

In BDBC (Fig. 1b), the first benzyl group A (C1–C6/C9) and the second benzyl group B (C15–C21) are planar with root mean square (r.m.s.) deviation of 0.0088 and 0.0142 Å, respectively with the dihedral A/B of 37.3 (8)°. The meta-positioned methoxy group C (C8/O2) is planar with plane of group A whereas *ortho*-positioned methoxy group D (C7/O1) is not planar with group A. The atoms of group C are at the distance of −0.0139 (4) and 0.0150 (3) Å, respectively from plane of group A whereas atoms of group D are at the distance of −1.1773 and 0.0631 Å, respectively from plane of group A. The cyclopentanone ring E (C11–C14/O3) is planar with r.m.s deviation of 0.0508 Å with dihedral angles E/A and E/B are 27.3 (7)° and 17.2 (1)°, respectively. The ring E is puckered with Pseudorotation parameters (*P* = 311.7 (6) Å, *θ* = 11.2(1)° for reference bond C10–C11) and adopts an exact envelope conformation on C14 atom. The molecules of BDBC are interlinked in the form of dimers through C–H⋯O bonding to complete R^2^_2_ (14) loop, the carbonyl O-atom of acts as H-bond acceptor for ortho-positioned CH of unsubstituted phenyl ring (Fig. 2b). The dimers are interlinked through C–H⋯O bonding, whereas the carbonyl O-atom of acts as H-bond acceptor for CH of five-membered ring. The crystal packing of BDBC is also further stabilized by weak C–H⋯π interaction that interlinked in the molecules in the form of infinite chain along *b*-axis (Table 2 and Fig. 3b). Off-set π⋯π stacking interaction also helps in the stabilization of the crystal packing with inter-centroid separation of 4.1625 (15) to 5.6812 (17) Å.

The cambridge structure data base search is performed for finding the related crystal structures from the literature. The search inferred that the crystal structures with reference codes OGAQUJ,^32^ QAKCIQ^33^ WAFJIY^34^ and ATUMEK^35^ have close resemblance with the crystal structure of BNTP. The crystal structure of OGAQUJ has unsubstituted phenyl rings, whereas QAKCIQ and WAFJIY have 4-hydroxyphenyl rings and 4-hydroxy-3,5-dimethoxy phenyl rings, respectively. Just like in crystal structure of BNTP, the tetrahydro-4*H*-pyran-4-one ring adopts envelope conformation in the related crystal structures. The nature of non-covalent interactions in the crystal structure of BNTP and OGAQUJ is same that is C–H⋯O, C–H⋯π and π⋯π stacking interactions. The O–H⋯O bonding is present in the crystal packing of QAKCIQ whereas O–H⋯O and C–H⋯O bonding are present in WAFJIY. π⋯π stacking interaction is absent in ATUMEK, QAKCIQ and WAFJIY. The search for the crystal structures related to the crystal structure of BDBC provides more than 40 hits but only one crystal structure has unsymmetrical substituted phenyl rings with reference code XEGSAG.^36^ One of the phenyl ring in XEGSAG is dimethylamino substituted whereas the other phenyl ring is cyano substituted. The crystal structures of XEGSAG and BDBC are crystallized in triclinic and orthorhombic crystal system, respectively. The cyclopentanone ring is almost planar in crystal structure of XEGSAG and BDBC. No H-bonding is present in crystal packing of XEGSAG and the molecules are interlinked by π⋯π stacking interaction whereas the crystal packing of BDBC is mainly stabilized by H-bonding. The bond lengths and bond angles of BNTP and BDBC (Table S1†) are consistent with the corresponding bond lengths and bond angles in the related crystal structures.

### Hirshfeld surface analysis

**Fig. 4:**
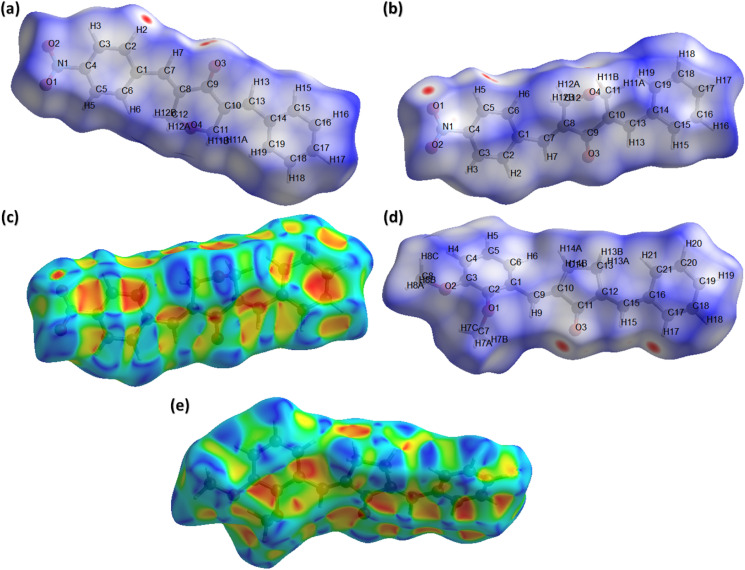
Hirshfeld surface (HS) plotted over dnorm for BNTP (a) view 1, (b) view 2. (c) Hirshfeld surface plotted over shape index for BNTP, (d) HS over dnorm for BDBC, (e) HS over shape index for BDBC.

In recent times, Hirshfeld surface analysis arouses as an economical and excellent analysis to explore the non-covalent interactions in the single crystals. The supramolecular behaviour in the single crystal can be explained by employing software named as Crystal Explorer 21.5.^37^ Hirshfeld surface can be plotted by using various properties like dnorm (normalized distances), shape index, curvedness *etc.* We are going to explore the information about the single crystals provided by Hirshfeld surface over dnorm and shape index. Red, blue and white colour are the main feather of the HS plotted over dnorm. Red and blue spots represent short and long contacts, respectively.^38–41^ White spots represent the contacts for which the center to center distance between the atoms is equal to the sum of the van der Waals radii of the atoms involved. Fig. 4a and b show HS plotted over dnorm for BNTP. Two views are shown as all the important short contacts of BNTP cannot be visible in a single view due to graphical limitations. The red spot around othro and meta CH of the phenyl ring (C1–C6) and carbonyl O-atoms indicates that these atoms are involved in H-boning. Fig. 4d is the HS plotted over dnorm for BDBC with atoms involved in short contacts are shown by red spot on the HS. HS plotted over shape index is a indictor weather π⋯π stacking interactions are present in the single crystal or not. The triangular regions of red and blue colour on this HS around aromatic rings indicate their involvement in the π⋯π stacking interactions.^42^ Such sort of regions are present in (Fig. 4c) and (Fig. 4e) indicates the presence of π⋯π stacking interactions but this interaction is weak as the inter-centroid separation between the interacting rings is greater than 4 Å for both compounds.

**Fig. 5:**
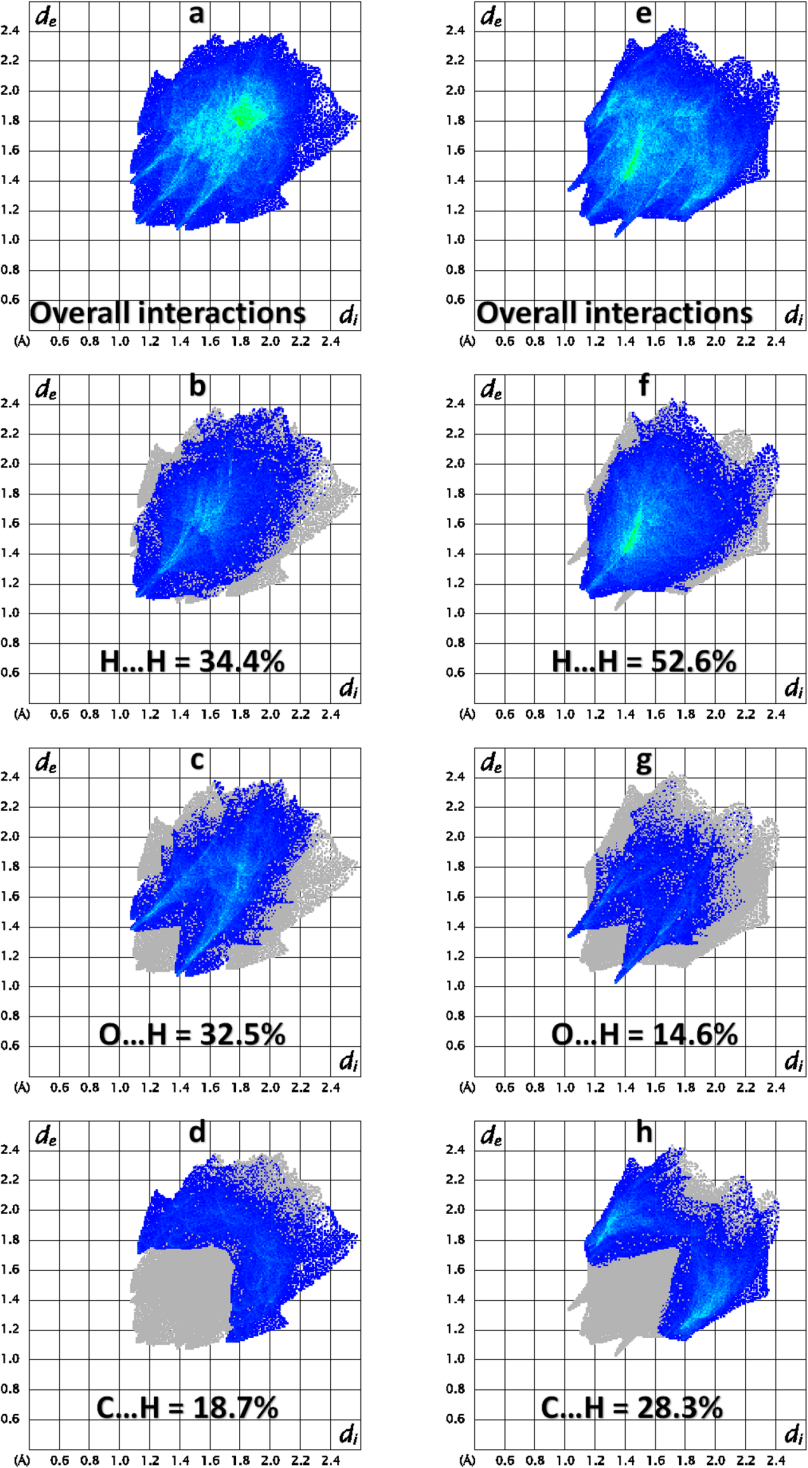
Important 2D finger print plots (a–d) for BNTP and (e–h) for BDBC.

2 D finger print plots (Fig. 5) are obtained by the keen investigation of the Hirshfeld surface that provides the contribution of each interatomic contact in the crystal packing.^43–46^ In 2 D plots, *d*_i_ is the distance from the HS to nearest atom inside it whereas *d*_e_ is the distance from the HS to nearest atom outside it. 2D finger print plot for all the possible interatomic contacts is shown by Fig. 5a and b for BNTP and BDBC, respectively. As C–H⋯O bonding is present in both compounds so the important conatcts for both compounds are H–H, O–H and C–H. For BNTP, the contribution of H–H, O–H and C–H interatomic contacts is 34.4%, 32.5% and 18.7%, respectively. For BDBC the contribution of H–H, O–H and C–H interatomic contacts is 52.6%, 14.6% and 28.3%, respectively. The contribution of O–H contact is larger in BNTP as compare to in BDBC. The reason behind is that the crystal structure of BNTP contains larger number of O-atoms than in BDBC. The same reason holds for the C–H contact. In the crystal packing of a single crystal, each interatomic contact has a unique ability or tendency to form the crystal packing interactions. For a particular crystal, some contacts are more favorable to form crystal packing interactions than the other contacts. Enrichment ratio for a contact provides the tendency of it to form crystal packing interactions.^47^ The contacts with enrichment ratio greater than one have higher tendency to form crystal packing interactions as compare to other contacts. The C⋯C contact is the most favourable contact in BNTP with enrichment ratio 2.11 (Table S2†) whereas the O⋯H contact is the most favourable contact in BDBC with enrichment ratio of 1.22 (Table S3†). The C⋯C contact is the not favourable contact in BDBC as the enrichment ratio for this contact is less than one.

**Fig. 6:**
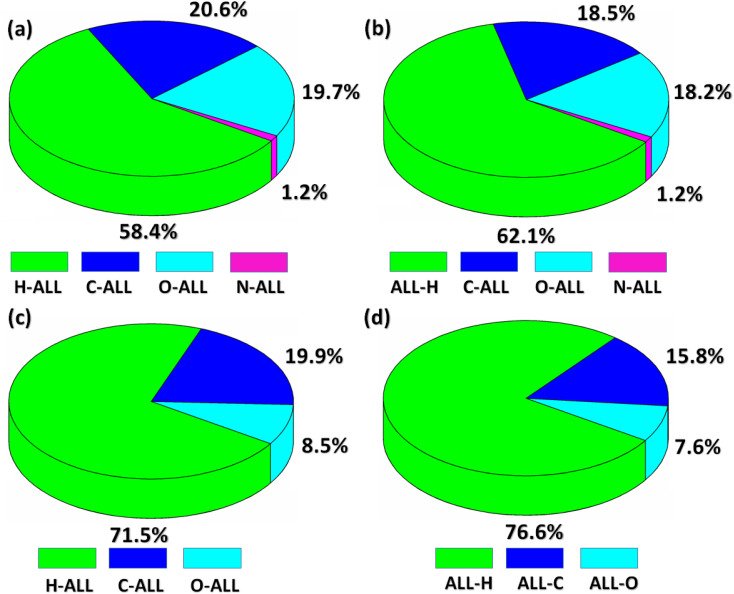
(a and c) Percentage contribution of the interaction of an atom inside of the HS with the atoms of molecules located in the neighboring of the HS for BNTP and BDBC. (b and d): Percentage contribution of the interaction of all the atoms inside of the HS with an atom of molecules located in the neighboring of HS for BNTP and BDBC.

Now, we are going to explore an interaction of atom present inside the HS with all the atoms present in the close vicinity of HS which we called as Atom-ALL interactions.^48^ For both compounds, H-ALL interaction has maximum contribution in the crystal packing. The percentage contribution of H-ALL interaction is 58.4% for BNTP (Fig. 6a**)** and 71.5% for BDBC (Fig. 6c). Similarly, we explore the ALL-atom interactions for both compounds that is the interaction of all the atoms present in the HS to an atom located in the close vicinity of the HS. ALL-H interaction is strongest for both compounds with value 62.1% for BNTP (Fig. 6b**)** and 76.6% for BDBC (Fig. 6d).

**Fig. 7:**
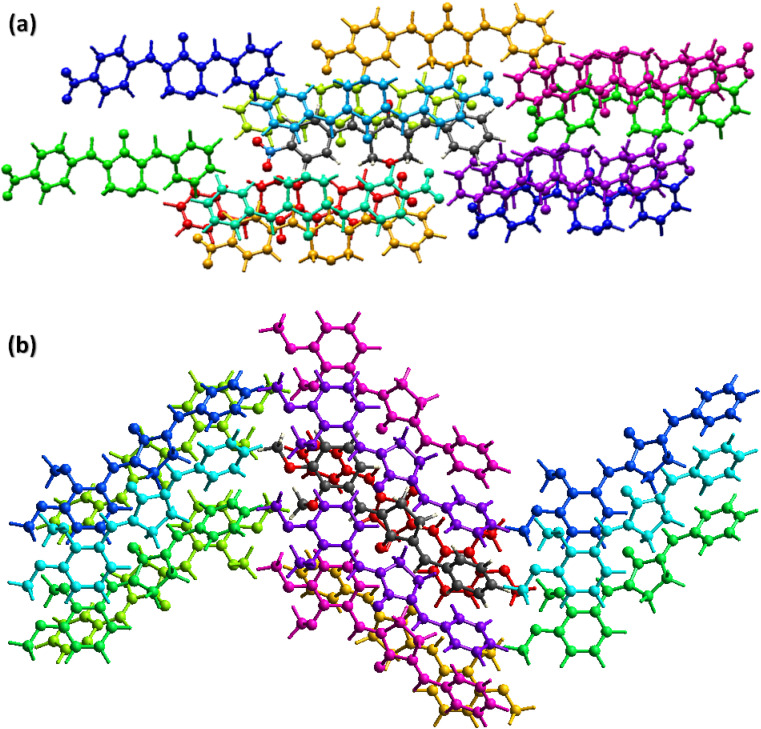
Interaction energy between the molecular pairs falling in the range of 3.8 Å of the reference molecule for (a), BNTP (b) BDBC.

**Fig. 8:**
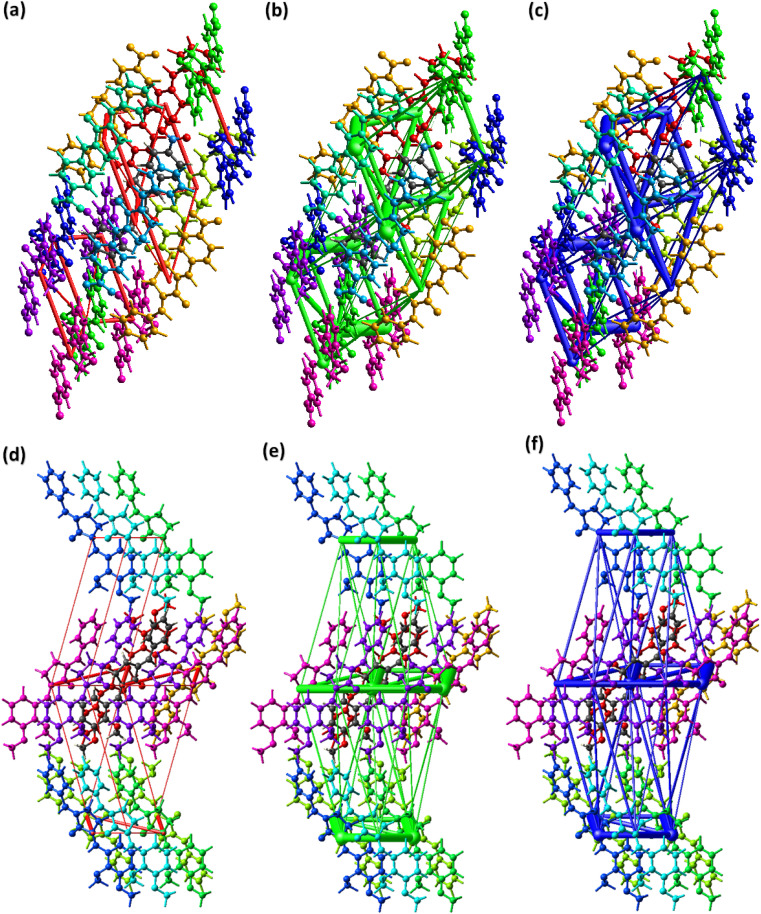
Energy framework in BNTP for (a) electrostatic energy, (b) repulsive energy, (c) total energy in. Energy framework in BDBC for (d) electrostatic energy, (e) repulsive energy, (f) total energy in BNTP.

The crystal packing environment is further explored in terms of finding the interaction energy between the pair of the molecules in both compounds. The calculations are done on the Crystal Explorer 21.5 software using built-in TONTO program. The accurate model named as B3LYP/6-31G(d,p) is used for the calculations. A cluster of molecules within 3.8 Å of the reference molecule is generated for the calculations of the interaction energy that is the sum of four kind of energies (electrostatic, polarization, dispersion and repulsion).^49–51^ The energy values are reported to a minimum value of 0.1 kJ mol^−1^ but the authors of Crystal Explorer program suggested that the reliability of the calculations is up to the minimum value of 1 kJ mol^−1^. The molecule containing dark gray C-atoms is the reference molecule for BNTP (Fig. 7a) and for BDBC (Fig. 7b). The scaled energies are listed in Table 3 for BNTP and Table 4 for BDBC. For the side by side interactions in both compounds, the major E_tot energies are (−34.1, −37, −54.3, −9.5, −25.3, −74.3, −11.5, −9.6, −1.2 kJ mol^−1^) for BNTP whereas the major E_tot energies (−79.9, −29.9, −11.5, −15.5, −13, −8.9, −41.1, −24.9 kJ mol^−1^) for BDBC. The electrostatic energy is mostly attractive for the pair of molecules but it may be repulsive for a pair of molecule. In the present case, the electrostatic energy is positive for two pair of molecules (4.5, 0.2 kJ mol^−1^) connected by (−*x* + 1/2, *y* + 1/2, −*z* + 1/2) in BNTP whereas for BDBC, the electrostatic energy is positive only for a single pair of molecules (0.8 kJ mol^−1^) connected by (*x*, *y* − 1/2, *z* + 1/2). The calculations inferred that the dispersion energy is the major contributor of the total attractive energy contribution for both compounds. In order to understand the topology of the single crystals, the energy frameworks are constructed in terms of joining the centroids of the interacting molecules by the cylinders are displayed in Fig. 7 (c and d) for electrostatic, repulsive and total energy, respectively which are obtained by using the interaction energy between the molecular pairs.^52^ The radius of the cylinder is proportion to the strength of the interaction. The cut off energy is set to be 5 kJ mol^−1^ and size of the cylinder is set to be 80 and cluster of molecules within 1 × 1 × 1 unit cell is used for generating the energy frameworks. For both compounds, the contribution of the repulsive energy is greater than the contribution of the electrostatic energy in defining the total energy as the radius of cylinders for repulsive energy framework is greater than the radius of the cylinder for electrostatic energy framework(Fig. 8).

**Table 3:** Interaction Energies (kJ mol^−1^) for BNTP. *R* is the distance between molecular centroids (mean atomic position) in Å. B3LYP/6-31G(d,p) electron density model is used for the calculations. The values of scale factors *k*_ele, *k*_pol, *k*_disp and *k*_rep are 1.057, 0.740, 0.871 and 0.618, respectively

*N*	Symmetry	*R*	*E*_ele	*E*_pol	*E*_dis	*E*_rep	*E*_tot
1	−*x*, −*y*, −*z*	7.11	−11.2	−2.9	−34.8	16.5	−34.1
2	*x*, *y*, *z*	7.59	−21.1	−6.1	−31.2	27.5	−37.0
1	−*x*, −*y*, −*z*	4.51	−13.3	−2.7	−68.5	34.6	−54.3
2	*x* + 1/2, −*y* + 1/2, *z* + 1/2	17.97	−2.7	−1.6	−6.3	0.0	−9.5
1	−*x*, −*y*, −*z*	6.74	−7.6	−4.2	−27.1	15.2	−25.3
1	−*x*, −*y*, −*z*	3.89	−21.4	−3.0	−96.9	56.6	−74.3
2	*x* + 1/2, −*y* + 1/2, *z* + 1/2	14.48	−2.3	−1.0	−9.6	0.0	−11.5
2	−*x* + 1/2, *y* + 1/2, −*z* + 1/2	14.42	4.5	−0.6	−15.9	0.0	−9.6
2	−*x* + 1/2, *y* + 1/2, −*z* + 1/2	18.67	0.2	−0.1	−1.5	0.0	−1.2

**Table 4:** Interaction Energies (kJ mol^−1^) for BDBC. *R* is the distance between molecular centroids (mean atomic position) in Å. B3LYP/6-31G(d,p) electron density model is used for the calculations. The values of scale factors *k*_ele, *k*_pol, *k*_disp and *k*_rep are 1.057, 0.740, 0.871 and 0.618, respectively

N	Symmetry	*R*	*E*_ele	*E*_pol	*E*_dis	*E*_rep	*E*_tot
1	−*x*, −*y*, −*z*	4.34	−24.1	−8.7	−101.3	65.0	−79.9
1	−*x*, −*y*, −*z*	8.45	−18.0	−9.8	−29.0	35.1	−29.9
2	−*x*, *y* + 1/2, −z + 1/2	13.71	−4.0	−0.3	−8.1	0.0	−11.5
2	*x*, −*y* + 1/2, *z* + 1/2	14.98	−5.9	−0.9	−9.9	0.0	−15.5
2	−*x* + 1/2, −*y*, *z*+1/2	15.09	−4.1	−0.6	−9.5	0.0	−13.0
2	*x*, −*y* + 1/2, *z* + 1/2	14.85	0.8	−0.7	−10.6	0.0	−8.9
2	−*x* + 1/2, *y* + 1/2, *z*	5.43	−5.9	−3.1	−60.6	32.7	−41.1
2	*x*, *y*, *z*	6.98	−3.5	−2.9	−35.0	18.5	−24.9

**Fig. 9:**
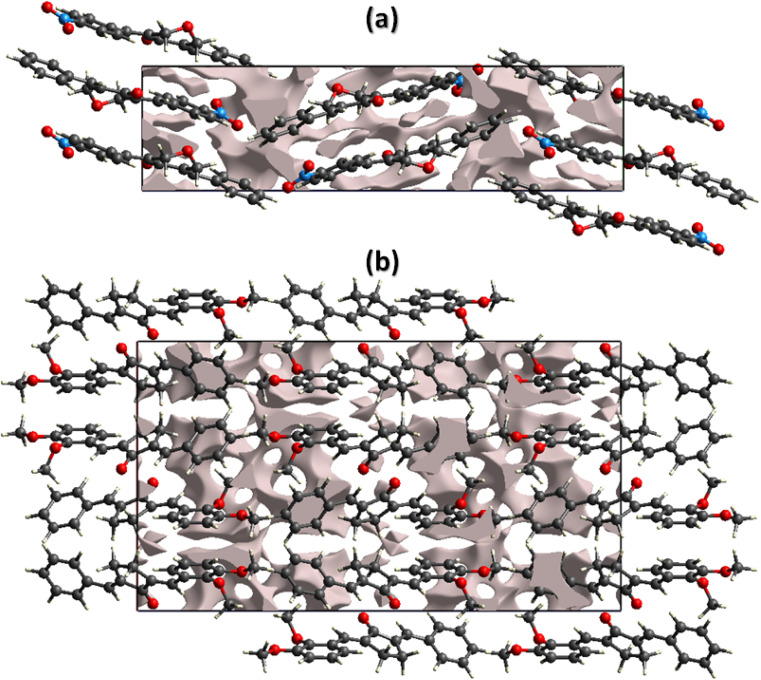
Graphical representation of voids in the crystal packing of (a) BNTP viewed along *a*-axis, (b) BDBC viewed along *b*-axis.

The mechanical strength of the single crystals mainly depends on the crystal packing. The single crystal with large cavities can bear only a small amount of external force whereas the single crystal with no large cavity can bear a significant amount of force or stress. By keeping in view the above said prospective, we performed void analysis for both compounds which is based on adding up the atomic electron density by using Hartree-Fock theory.^53–55^ It is assumed that all the atoms are spherically symmetric while calculating voids. The void volume is 149.02 Å^3^ for BNTP and 273.41 Å^3^ for BDBC. The volume occupied by the voids is 9.72% in BNTP and 8.36% in BDBC. As the voids occupy very small space in both compounds so it indicates that there is no large cavity in the crystal packing of both compounds and both compounds are expected to have good mechanical properties(Fig. 9).

### Computational methodology

All quantum chemical calculations were achieved through the Gaussian 16 program.^56^ The molecular geometries of BNTP and BDBC were performed with M06-2X functional and 6-311G* basis set. Among the conventional DFT functionals, the results for hyperpolarizability calculations using M06-2X has been found satisfactory in previous several studies.^57^ All default convergence criteria are used for current calculations. The optimized geometries were subsequently subjected to frequency calculations. The absence of any negative frequency confirms the global minimum of our optimized structure on its potential energy surface. The UV-Visible spectrum was calculated using time dependent (TD) M06-2X method with same basis set 6-311G*. The linear and nonlinear optical polarizabilities were calculated using Polar keyword. Our earlier pure computational studies shows more details^58–60^ to calculate nonlinear (NLO) responses of organic molecules using Kohn–Sham perturbation method through Gaussian 16.^61^ It is possible to determine the average third-order nonlinear polarizability (*γ*), isotropic (<*α*>) and anisotropic linear polarizability (Δ*α*), and dipole moment *μ*, of the molecules using the following formulas.

1 *μ* = (*μ*_*x*_^2^ + *μ*_*y*_^2^ + *μ*_*z*_^2^)

The average isotropic polarizability (*α*_iso_) can be calculated by following equations:2
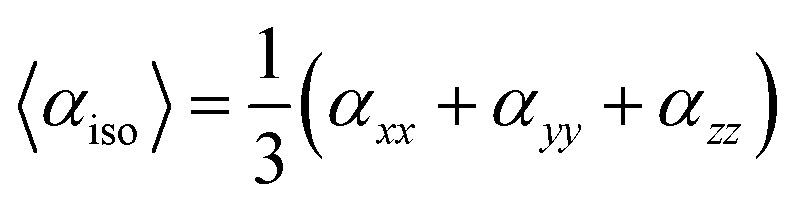


For anisotropy of polarizability (Δ*α*) and average static second hyper polarizability, these can be calculated by following equations:3
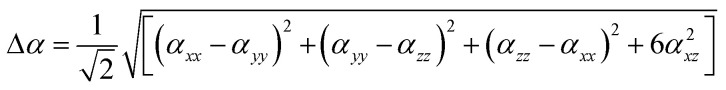
4
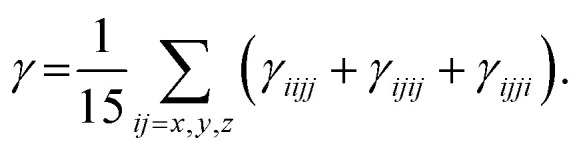
Under Kleinman symmetry,5
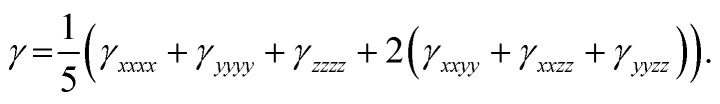


### Molecular geometries

**Fig. 10:**
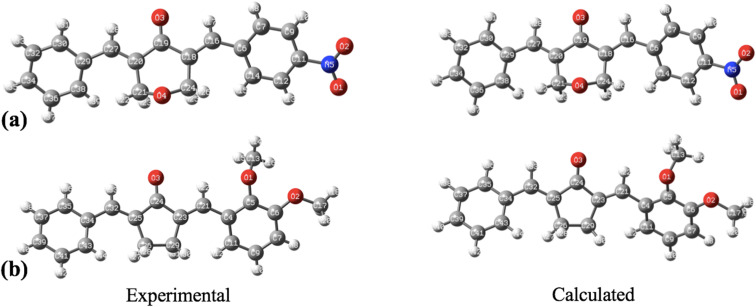
The geometrical comparison of experimental and calculated structures of (a) BNTP and (b) BDBC.

**Fig. 11:**
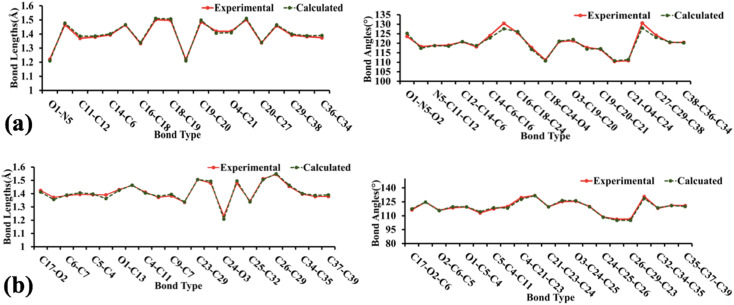
The graphical representation of experimental and calculated bond lengths and bond angles of BNTP (a) and BDBC (b).

The geometry optimization, frequency, and all other computational calculations of BNTP and BDBC compounds have been carried out by employing M06-2X functional and 6-311G* functional basis set. The experimental geometry and theoretically most stable geometry of BNTP and BDBC compounds are shown in Fig. 10. This theoretically stable geometry was used for determination of optimized geometrical parameters (bond lengths and bond angles) along with non-linear optical properties of these molecules (linear polarizability and first order hyper polarizability). The optimized structural parameters are compared with experimentally determined XRD structure. These parameters represent a good approximation. The graphical comparison of experimental and computed structures of both BNTP and BDBC are shown in Fig. 11, it illustrates that although theoretical calculations of bond lengths and bond angles performed in gaseous phase, they are in good agreement with experimental values (in solid phase). In BNTP, noticeable difference is observed between the theoretical and experimental bond angles between C6–C16–C18 and C20–C27–C29, that is ∼3 Å and 2 Å because in substituted benzene, the carbon atoms of the ring exert a large attraction on valence electronic cloud.

### Linear polarizability

The most basic electric response qualities are linear polarizability, and the calculation of these values has provided an excellent foundation for evaluating the accuracy of electronic state calculations with various theoretical models in the field of quantum chemistry.^62^Table 5 shows the average isotropic *α*_iso_ and anisotropic polarizabilities along with their individual components for compounds BNTP and BDBC at M06-2X functional and 6-311G* basis set. The isotropic polarizabilities of compound BNTP and BDBC are 48.10 × 10^−24^ esu and 51.85 × 10^−24^ esu respectively, which shows that the value of isotropic polarizability of BDBC is slightly (3.75 × 10^−24^ esu) greater than BNTP, while the anisotropic polarizability values of BNTP and BDBC have only a small difference of 1.08 × 10^−24^ esu. Among the individual components, *α*_*zz*_ shows maximum value of linear polarizability in both BNTP and BDBC molecules as 79.35 × 10^−24^ esu and 73.03 × 10^−24^ esu, respectively, which shows that polarization and ICT occurred mainly along *z*-axis. The linear isotropic polarizability *α*_iso_ and anisotropic Δ*α* for BNTP and BDBC are about similar, which indicates direction effects are negligible during induced polarization.

**Table 5:** The average isotropic and anisotropic polarizabilities (×10^−24^ esu) along with their individual components for compounds BNTP and BDBC at M06-2X/6-311G* levels of theory

α components	BNTP	BDBC
*α*_*xx*_	21.43	41.06
*α*_*xy*_	1.089	−14.46
*α*_*yy*_	43.53	32.74
*α*_*xz*_	−0.030	14.28
*α*_*yz*_	43.72	−0.743
*α*_*zz*_	79.35	73.037
*α*_iso_	48.10	51.85
Δ*α*	51.22	50.14

### Third-order NLO Polarizability

Besides linear polarizability and second-order NLO polarizability, third-order NLO polarizability of BNTP and BDBC compounds have been also calculated at the same M06-2X/6-311G* levels of theory. The third order non-linear polarizability *γ* is characteristic of two photon absorption phenomena. For second order hyperpolarizability, along with individual tensors, we have also calculated average third order polarizability(<*γ*>) that are shown in Table 6, the average static third order nonlinear polarizability <*γ*> of BNTP and BDBC compounds are considerately larger mounting to 226.45 × 10^−36^ esu and 238.72 × 10^−36^ esu, respectively, which indicate their relatively higher NLO response. The average second-order NLO polarizability of compound BDBC is larger than BNTP. It will be also interesting to make a comparative analysis of <*γ*> amplitudes of our synthesized compounds to some other standard and previously known molecules. For this purpose, we have also calculated the <*γ*> amplitude of *para*-nitroaniline (*p*-NA), which is a typical push–pull organic compound and has been used as a reference molecule in several investigations. The <*γ*> amplitude of *p*-NA was found to be (8.02 × 10^−36^ esu at the M06-2X/6-311G* level of theory in our previous investigation.^63^ A semi-quantifiable comparison illustrates that BNTP and BDBC compounds possess the <*γ*> amplitudes, which are approximately 28 and 30 times larger than that of *p*-NA,respectively, as calculated using same methodology. Besides this, the <*γ*> amplitudes of BNTP and BDBC compounds are also comparable to previously studied NLO molecules, *e.g.* oxalate compound (40.64 × 10^−36^ esu at M06/6–31 + G) studied by Essid *et al.*;^64^ acyl thiourea BTCC derivative (27.30 × 10^−36^ esu at M06-2X/6-311G*) studied by Ashfaq *et al.*;^65^ donor–acceptor chalcone derivatives (79.31 × 10^−36^ esu at PBE0/6-311G**) studied by Muhammad *et al.*,^60^ and also better than calculated <*γ*> amplitudes for C60 fullerene and its different heteroatom doped molecules.^66^ This comparison indicates a semi-quantitative insight for the real-time potential of our indigenously synthesized molecules in NLO applications.

**Table 6:** The calculated values of third-order polarizability *γ* (×10^−36^ esu) along with its individual components as for BNTP and BDBC compounds at M06-2X/6-311G* levels of theory

*γ*	BNTP	BDBC
*γ*_*xxxx*_	6.500	106.08
*γ*_*yyyy*_	174.78	23.154
*γ*_*zzzz*_	892.88	549.19
*γ*_*xxyy*_	4.757	93.463
*γ*_*xxzz*_	47.386	144.59
*γ*_*yyzz*_	55.592	19.506
<*γ*>	226.45	238.72

Regarding the origin of relatively larger NLO polarizabilities, it is seen that both entitled molecules illustrate a decent intramolecular charge transfer character. It is important to pen down that origin of <*γ*> amplitudes can be traced from wildly used three-state approximation^67^ by means of optical parameters as calculated by TD-DFT method and given in Table 7. Assuming the three-state approximation, a crude approximation and only longitudinal dominant component which is *γ*_*zzzz*_ in current calculations can be correlated with optical transition parameters. According to three-state approximation, a lower transition energy with larger oscillator strength and larger change in dipole moment between ground to excited states can cause a larger *γ*_*zzzz*_ amplitude. The *γ*_*zzzz*_ amplitudes of BNTP and BDBC compounds are found to be 892.88 × 10^−36^ esu and 549.19 × 10^−36^ esu, respectively. The larger amplitude of *γ*_*zzzz*_ for BNTP might be attributed to the lower energy transitions (S_0_ to S_1_ and S_0_ to S_3_) in BNTP as compared to BDBC compounds as shown in Table 7.

**Table 7:** The Transition energies (Δ*E*) in eV, oscillator strength (*f*_o_), excited dipole moment Δ*μ*, and percentage configuration interactions (C.I) of BNTP and BDBC molecules calculated at M06-2X/6-311G* level of theory

Molecules	Electronic excitation	Δ*E*	*f*_o_	Δ*μ*	Major contribution	% C.I
BNTP	S_0_ → S_1_	3.356	0.0078	0.307	H-3 → L	55
	S_0_ → S_2_	4.0188	0.0034	0.186	H → L	55
	S_0_ → S_18_	4.177 (3.814)^a^	1.1770	3.387	H → L	55
BDBC	S_0_ → S_2_	4.074 (3.646)^a^	1.2350	3.514	H → L	68
	S_0_ → S_3_	4.409	0.0293	0.3604	H-1 → L + 1	60

a The values in the parenthesis are experimentally obtained and compared with the most intense calculated peaks (the highest oscillator strength) in entitled molecules.

### The UV-visible spectra

**Fig. 12:**
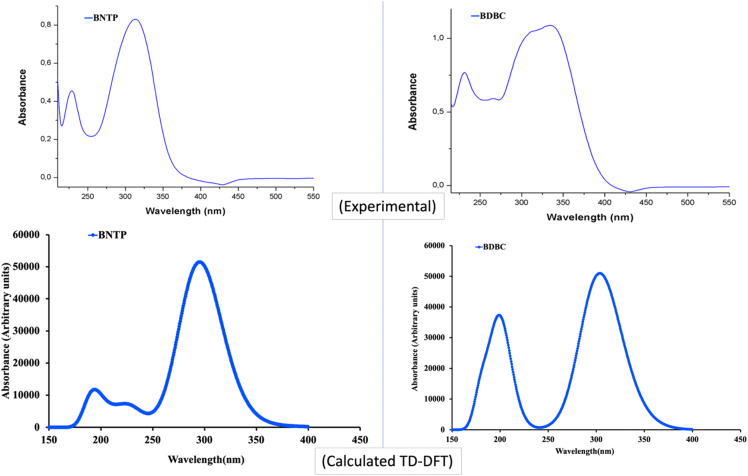
The Experimental and calculated (M06-2X/6-311G* levels) absorption spectra of BNTP and BDBC compounds.

**Fig. 13:**
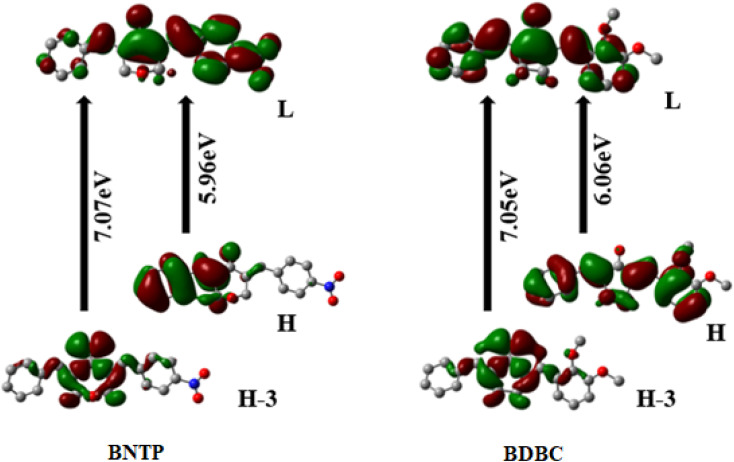
Frontier molecular orbitals (FMOs) of BNTP and BDBC compounds H atoms were omitted for clarity.

The UV– isible spectrum offers useful information on the electronic structure, optical and photovoltaic properties of the molecule. The absorption peaks in the optical spectrum frequently involve the transfer of electrons from σ, *n* and π orbitals of the ground state to the σ* and π* orbitals of higher excited states.^68^ The absorption spectrum of both molecules were obtained through time-dependent-DFT calculations as well as through experimental analysis as shown in Fig. 12. The molecule BNTP exhibits two intense absorption peaks at 296 nm with oscillator strength of 1.177 and another peak of lower intensity at 193 nm, which are experimentally observed around 320 nm and 175 nm, respectively, as shown in Fig. 12. The calculated absorption peaks of 193 nm and 296 nm can be attributed as *n* to π* and π to π* transitions, respectively, as perceived from Fig. 13 of FMOs. Similarly, BDBC molecule also illustrates two important absorption peaks that is at 304 nm with oscillator strength of 1.235 and also with relative less intense peak at 198 nm. The absorption peaks of BDBC molecule are relatively broader and slightly red shifted as compared with absorption peaks of BNTP molecule. Both compounds did not show any significant absorption above 400 nm, and their relative transparency is an advantageous factor for efficient NLO applications.

The most important orbitals in the molecule are the lowest-lying unoccupied molecular orbital (LUMO) and the highest occupied molecular orbital (HOMO), also known as frontier molecular orbitals (FMO). The FMOs play a crucial role in molecule interactions as well as molecule electronic spectra.^69–71^ The way of interaction of the molecule with other is determined by these orbitals. The kinetic stability and chemical reactivity of a molecule can be determined using the energy gap between frontier orbitals. A molecule with a small frontier orbital gap is more polarizable and is often associated with low kinetic stability and strong chemical reactivity.^72^ The lower value between HOMO and LUMO energy gap in BNTP than BDBC (5.96 eV and 6.06 eV, respectively) makes it less stable and more reactive. The LUMO orbital primarily act as electron acceptor while HOMO is the orbital that mostly act as an electron donor. The frontier molecular orbitals of BNTP and BDBC compounds are shown in Fig. 13 In case of BNTP molecules, LUMO is spread towards NO2 group side and in BDBC, LUMO is spread over whole compound. While in case HOMO-3, orbitals accumulate at the central region of both compounds.

### Molecular electrostatic potential analysis

**Fig. 14:**
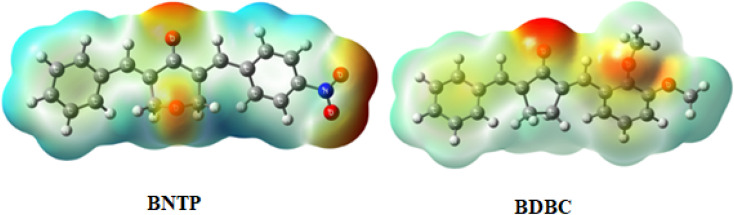
Molecular electrostatic potential diagram (MEP) of BNTP and BDBC.

The interaction of energy between electrons of the molecule produced from the electrical charge and a unit positive charge is used to describe the molecular electrostatic potential in the environment around the molecule. The molecular electrostatic potential (MEP) is related to electron density and is a useful tool for determining where electrophilic and nucleophilic assaults. It also enables to visualize the size, charge density, shape, and charge related properties of molecules. To find the reactive sites for nucleophilic and electrophilic attacks, MEP of BNTP and BDBC compounds were draw and shown in Fig. 14. In compound BNTP, most of the negative potential is because of the presence of oxygen atoms of terminal nitro group, and this is the site of electrophilic attack. Moreover, oxygens atoms present on the central ring also exhibits negative potential. The blue color indicates the positive potential perhaps created by the withdrawing effect of nitro group, undergoing nucleophilic attack. In compound BDBC, most of the negative potential is due to terminal methoxy group that leads to electrophilic attack while electronic charge density delocalized due to conjugated pi bonds shown by slight blue color that is susceptible to nucleophilic attack. However, a careful analysis of MEPs of both compounds shows that BNTP compound is more susceptible to electrophilic attack as compared to that of BDBC compound.

## Conclusion

The crystalline unsymmetrical diarylidene alkanones are synthesized *via* the Aldol condensation reaction. The title compounds BDBC and BNTP are characterized by the single crystal x-rays diffraction and NMR analysis. Crystal packing of both compounds are stabilized by C–H⋯O, C–H⋯π and π⋯π stacking interactions. The intermolecular interactions are comprehensively explored by Hirshfeld surface analysis. In order to explore the topology of BDBC and BNTP, the interaction energy between molecular pairs are calculated and energy frameworks are constructed. Void analysis inferred that there is no large cavity in the crystal packing of both compounds. The quantum chemical computations were also successfully applied on the synthesized molecules. The geometries were optimized to the lowest energy ground states and compared with their counterpart experimental bonding parameters. The optimized geometries were further used for the calculation of optical and NLO properties. The average static third order nonlinear polarizability <*γ*> of BNTP and BDBC compounds are considerately larger mounting to 226.45 × 10^−36^ esu and 238.72 × 10^−36^ esu, respectively. A semi-quantitative evaluation illustrates that BNTP and BDBC compounds possess the <*γ*> amplitudes, which are approximately 28 and 30 times larger than that of *p*-NA, respectively, as calculated using same methodology. The non-zero amplitudes of third-order NLO polarizabilities indicate that entitled molecules possess decent potential for their future applications for optoelectronic and NLO applications.

## Author contributions

Akbar Ali: supervision, writing – original draft, data curation. Shabbir Muhammad: DFT study, Software, supervision, methodology. Zia Ud Din: data curation, writing – original draft preparation. Muhammad Ibrahim: statistical analysis, data curation, writing – original draft preparation. Abdullah G. Al-Sehemi: methodology, software conceptualization. Muhammad Ashfaq & Muhammad Nawaz Tahir: writing – reviewing and editing, resources of the SC-XRD part. Edson Rodrigues-Filho: supervision, resources. Dania Gull: methodology, writing – original draft preparation, Muhammad Suleman: reviewing, editing, UV and IR data curation.

## Conflicts of interest

All authors agree to declare no conflict of interest regarding any content or publication of this work in the form of a manuscript.

## Supplementary Material

RA-013-D2RA07681K-s001

RA-013-D2RA07681K-s002
