# Supplementary material for: Acid catalyzed one-pot approach towards the synthesis of curcuminoid systems: unsymmetrical diarylidene cycloalkanones, exploration of their single crystals, optical and nonlinear optical properties
Source: RSC Adv. 2023 Feb 2;13(7):4476–94. doi: 10.1039/d2ra07681k (PMC9892888; doi:10.1039/d2ra07681k)
Supplement: RA-013-D2RA07681K-s001 [file RA-013-D2RA07681K-s001.pdf]

Electronic Supplementary Material (ESI) for RSC Advances.
This journal is © The Royal Society of Chemistry 2023

Acid catalyzed one-pot approach towards the synthesis of curcuminoids systems: unsymmetrical diarylidene cycloalkanones, exploration of their single crystals, optical and nonlinear optical properties

Akbar Ali,[1] Zia Ud Din,[2] Muhammad Ibrahim,*[3] Muhammad Ashfaq,*[4] Shabbir Muhammad,[5] Dania Gull,[1] Muhammad Nawaz Tahir,[4] Edson Rodrigues-Filho,[2] Abdullah G. Al-Sehemi,[5] Muhammad Suleman[6]

[1]Department of Chemistry, Government College University Faisalabad, 38000-Faisalabad, Pakistan.

[2]LaBioMMi, Departamento de Química, Universidade Federal de São Carlos, CP 676, São Carlos, SP 13.565-905, Brazil

[3]Department of Applied Chemistry, Government College University Faisalabad, Pakistan.

[4]Department of Physics, University of Sargodha, Sargodha, Pakistan

[5]Department of Chemistry, College of Science, King Khalid University, P.O. Box 9004, Abha 61413, Saudi Arabia

[6]Department of Chemistry, Riphah International University Faisalabad Campus, Pakistan

* Corresponding authors E-mail addresses:

(Dr. Muhammad Ibrahim) ibrahimchem@gmail.com

(Muhammad Ashfaq) ashfaq.muhammad@uos.edu.pk;muhammadashfaq1400@gmail.com

Table: S1 Selected bond lengths and bond angles in **BNTP-BDBC**.

Selected bond lengths in **BNTP**		Selected bond lengths in **BDBC**	
O1-N1	1.2209 (18)	O1-C2	1.391 (3)
O2-N1	1.2156 (18)	O1-C7	1.430 (3)
N1-C4	1.4666 (18)	O2-C3	1.373 (3)
O3-C9	1.2185 (18)	O2-C8	1.424 (3)
O4-C11	1.4197 (18)	O3-C11	1.232 (3)
O4-C12	1.4204 (17)	-	-
Selected bond angles in **BNTP**		Selected bond angles in **BDBC**	
O1-N1-O2	123.58 (14)	C2-O1-C7	113.19 (18)
O1-N1-C4	118.31 (14)	C3-O2-C8	116.5 (2)
O2-N1-C4	118.10 (13)	C3-C2-O1	118.8 (2)
C8-C9-O3	120.85 (13)	C4-C3-O2	124.8 (2)
C11-O4-C12	110.80 (12)	C10-C11-O3	125.3 (2)

Table S2 Enrichment ratio for the pair of interatomic contacts in **BNTP**. The enrichment ratio is not calculated for the contacts having random contacts less than 0.9% as these contacts have negligible contribution in the crystal packing.

**Contact %**	**Atom**	**H**	**C**	**N**	**O**
	**H**	34.4	18.7	0.4	32.5
	**C**	18.7	8.1	1	3.3
	**N**	0.4	1		1
	**O**	32.5	3.3	1	0.6
**Surface%**		60.2	19.6	1.2	19
**Random Contacts %**	**Atom**	**H**	**C**	**N**	**O**
	**H**	36.24			
	**C**	23.60	3.84		
	**N**	1.44	0.47	0.01	
	**O**	22.88	7.45	0.46	3.61
**Enrichment ratio**	**Atom**	**H**	**C**	**N**	**O**

	**H**	0.95			
	**C**	0.79	2.11		
	**N**	0.28			
	**O**	1.42	0.44		0.17

Table S3 Enrichment ratio for the pair of interatomic contacts in **BNTP**. The enrichment ratio is not calculated for the contacts having random contacts less than 0.9% as these contacts have negligible contribution in the crystal packing.

**Contact %**	**Atom**	**H**	**C**	**O**
	**H**	52.6	28.3	14.6
	**C**	28.3	3	1.5
	**O**	14.6	1.5	
**Surface%**		74.05	17.9	8.05
**Random Contacts %**	**Atom**	**H**	**C**	**O**
	**H**	54.83		
	**C**	26.51	3.20	
	**O**	11.92	2.88	0.65
**Enrichment ratio**	**Atom**	**H**	**C**	**O**
	**H**	0.96		
	**C**	1.07	0.94	
	**O**	1.22	0.52	

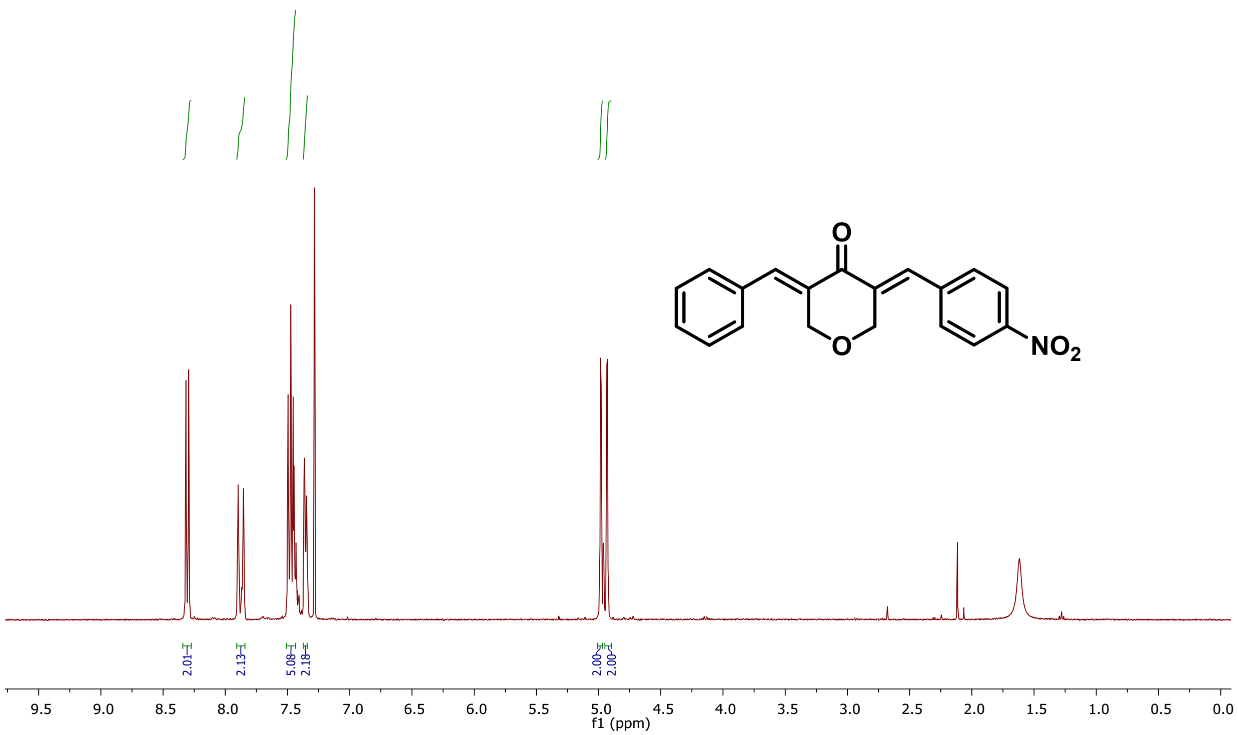

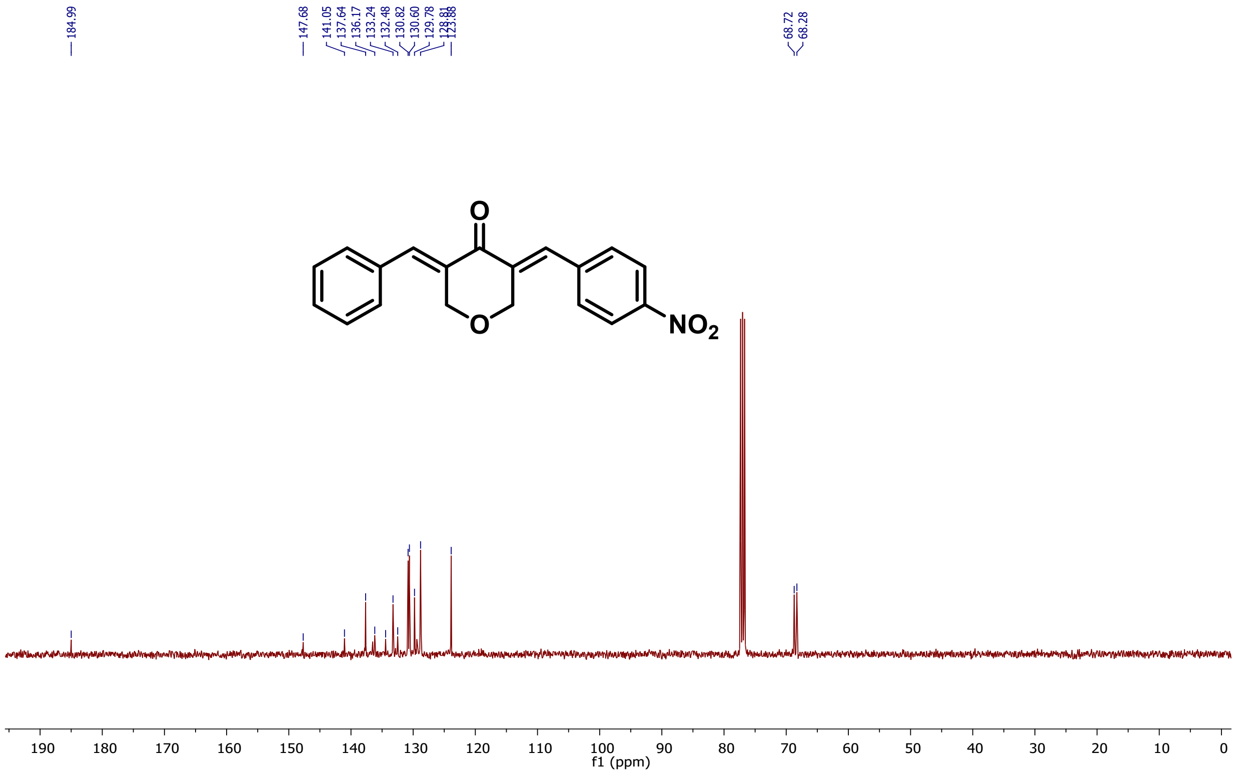

Fig. S1 ^{1}H and ^{13}C NMR data of the compound **BNTP.**

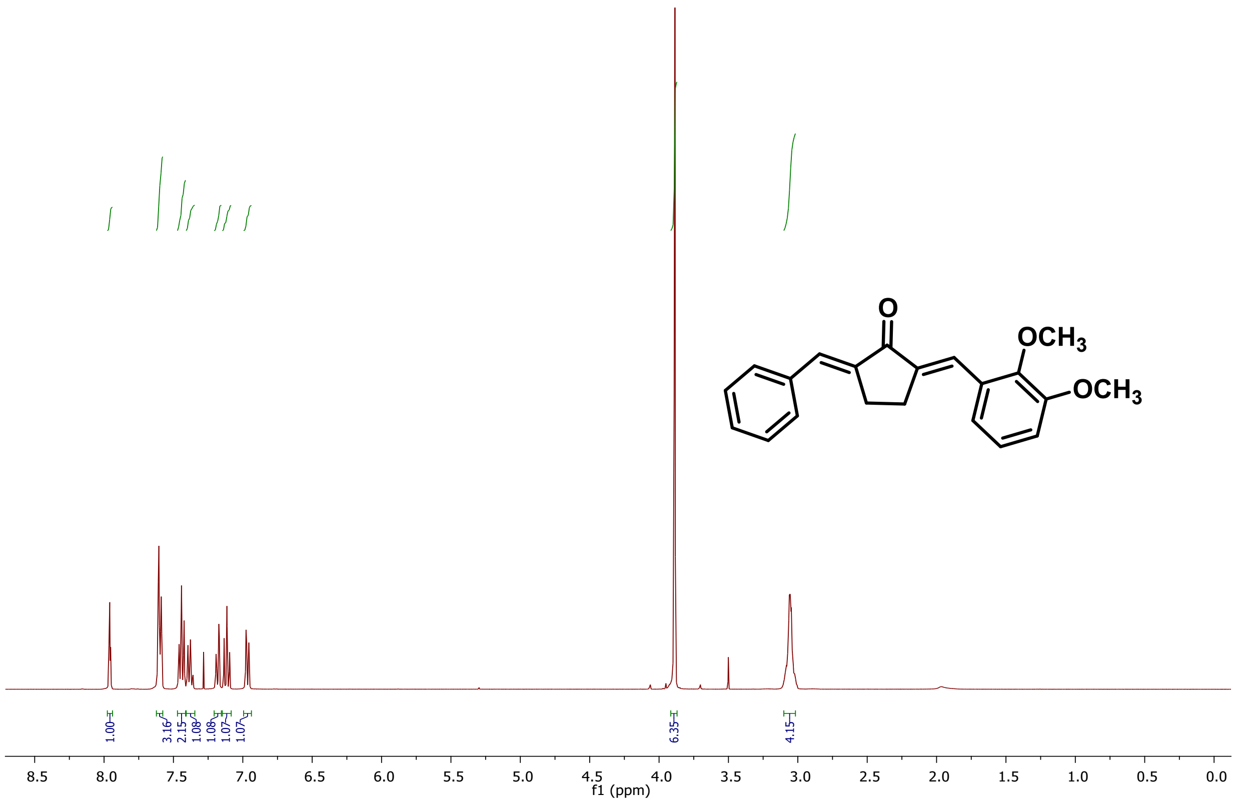

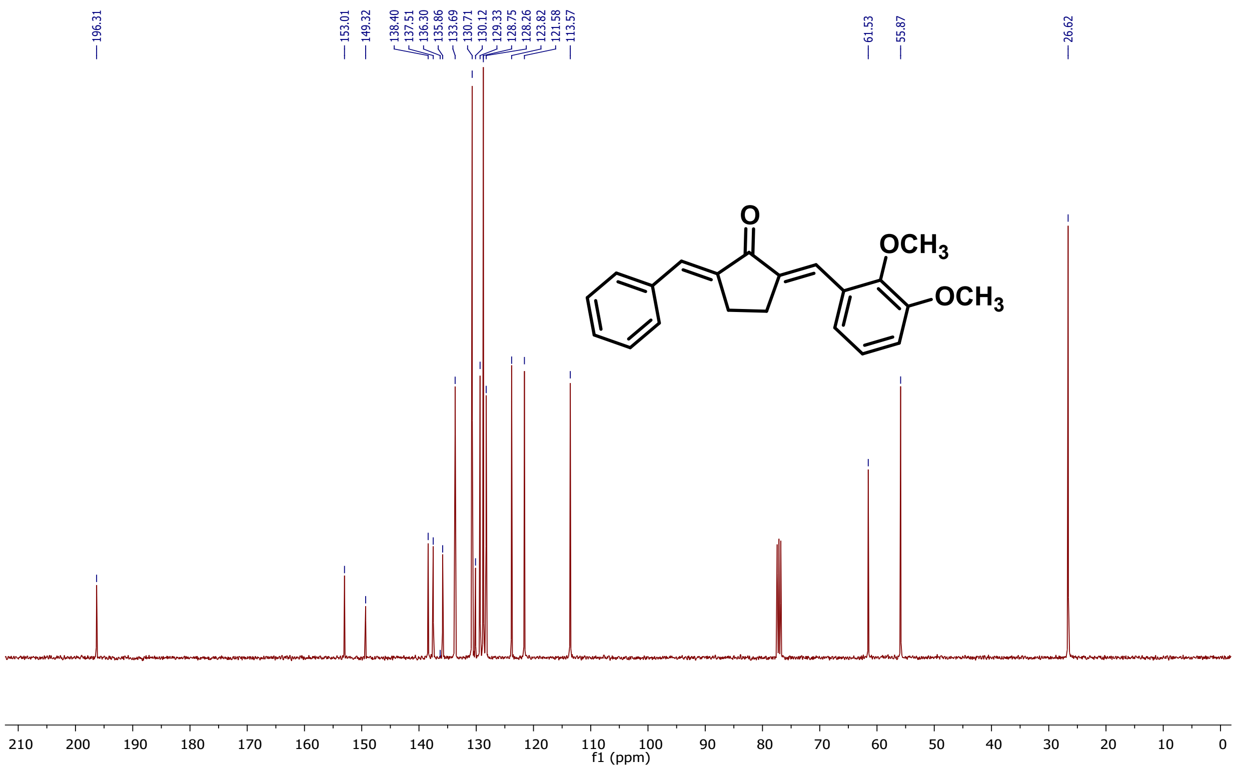

Fig.S2 ^{1}H and ^{13}C NMR data of the compound **BDBC.**

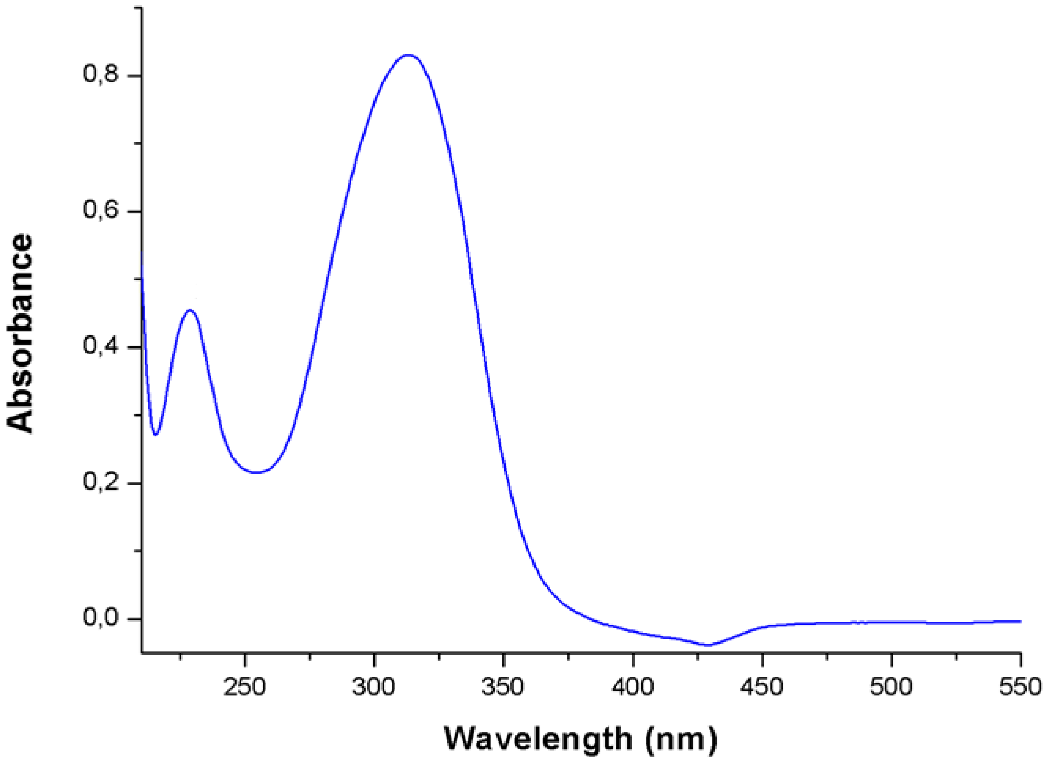

Fig. S3 UV spectrum of the compound **BNTP.**

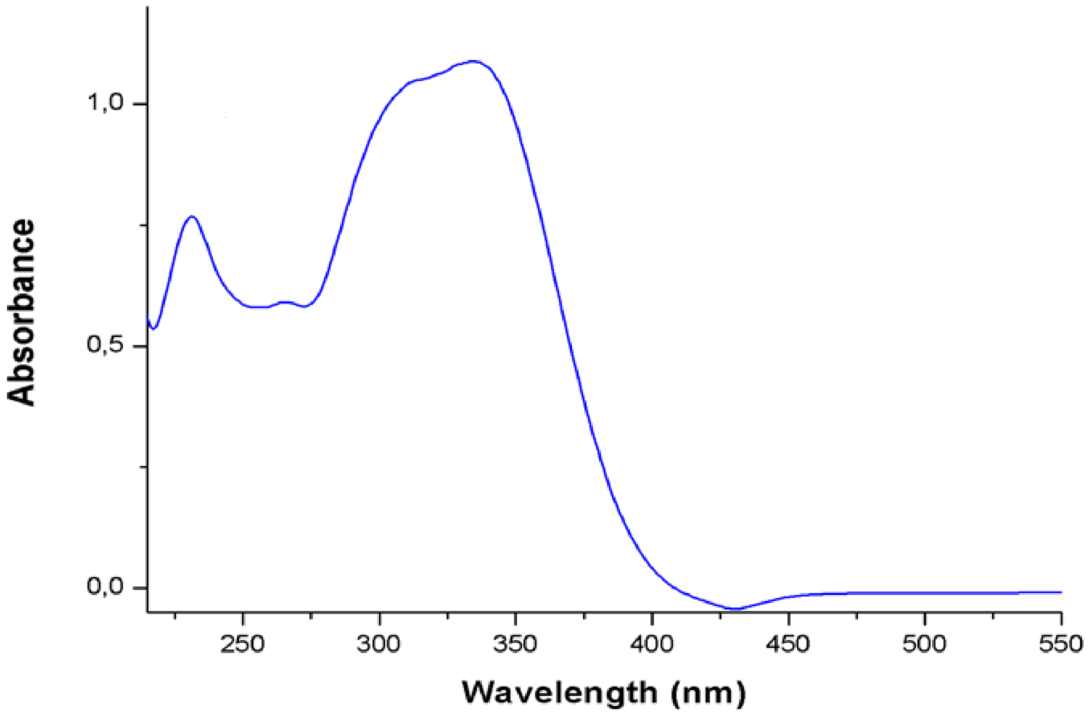

Fig. S4 UV spectrum of the compound **BDBC.**

IR-Prestige21

Functional Groups IR values

1. 3053 cm^{-1} (=C-H)
2. 2833 cm^{-1}, 2924 cm^{-1} (=C-H) Antisymmetry, Symmetry
3. 1670 cm^{-1} (C=O)
4. 1612 cm^{-1} (C=C)
5. 1492 cm^{-1} (-CH_2)
6. 1444 cm^{-1} (-NO_2)
7. 1265 cm^{-1} (C-O)

Comment:
A1K7iA11

Nº of Scans: 64
Resolution: 4.0
Apodization: Happ-Genzel

Date/Time: 14/12/2022 09:23:16
User: LV

SHIMADZU

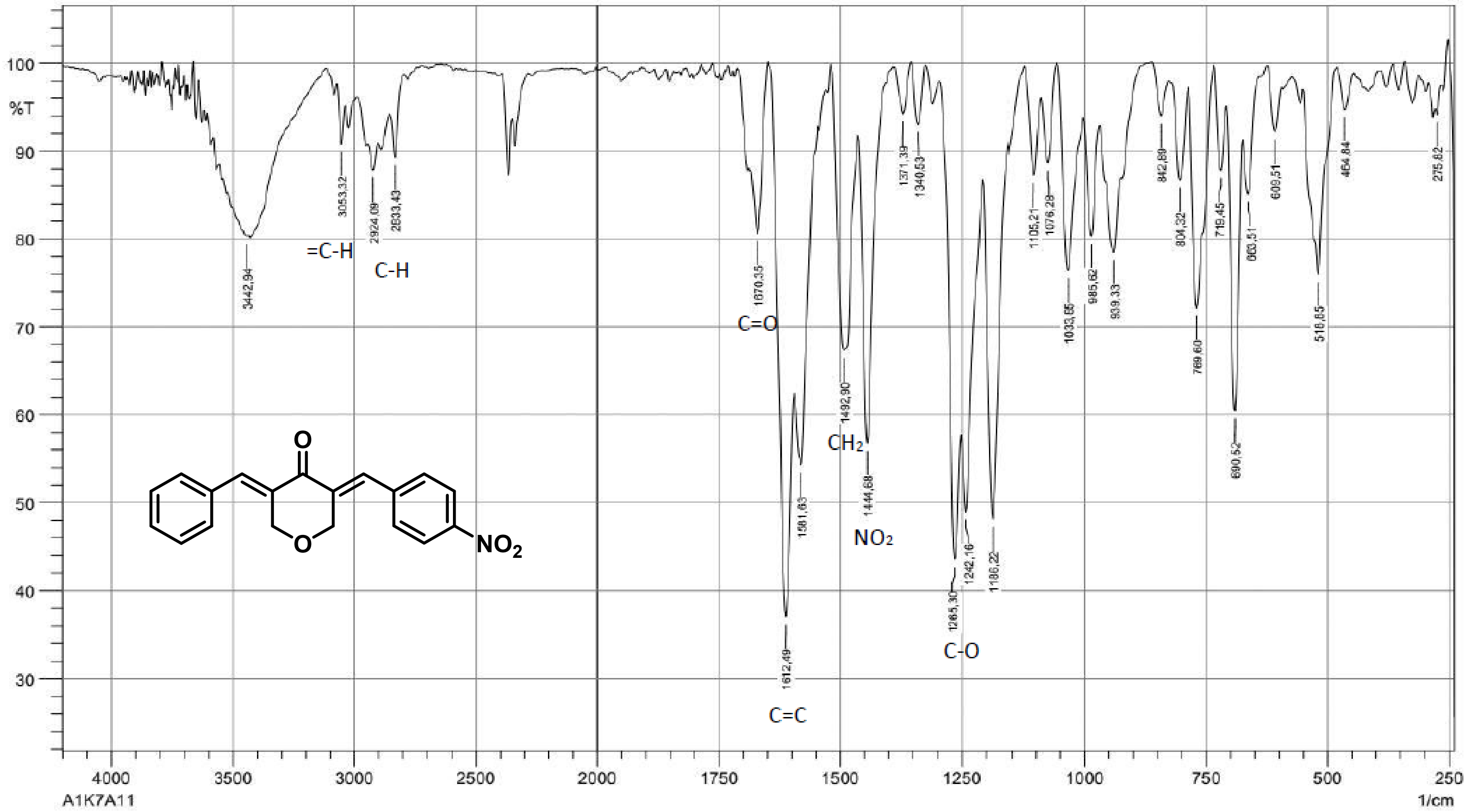

Fig. S5 IR spectrum of the compound **BNTP.**

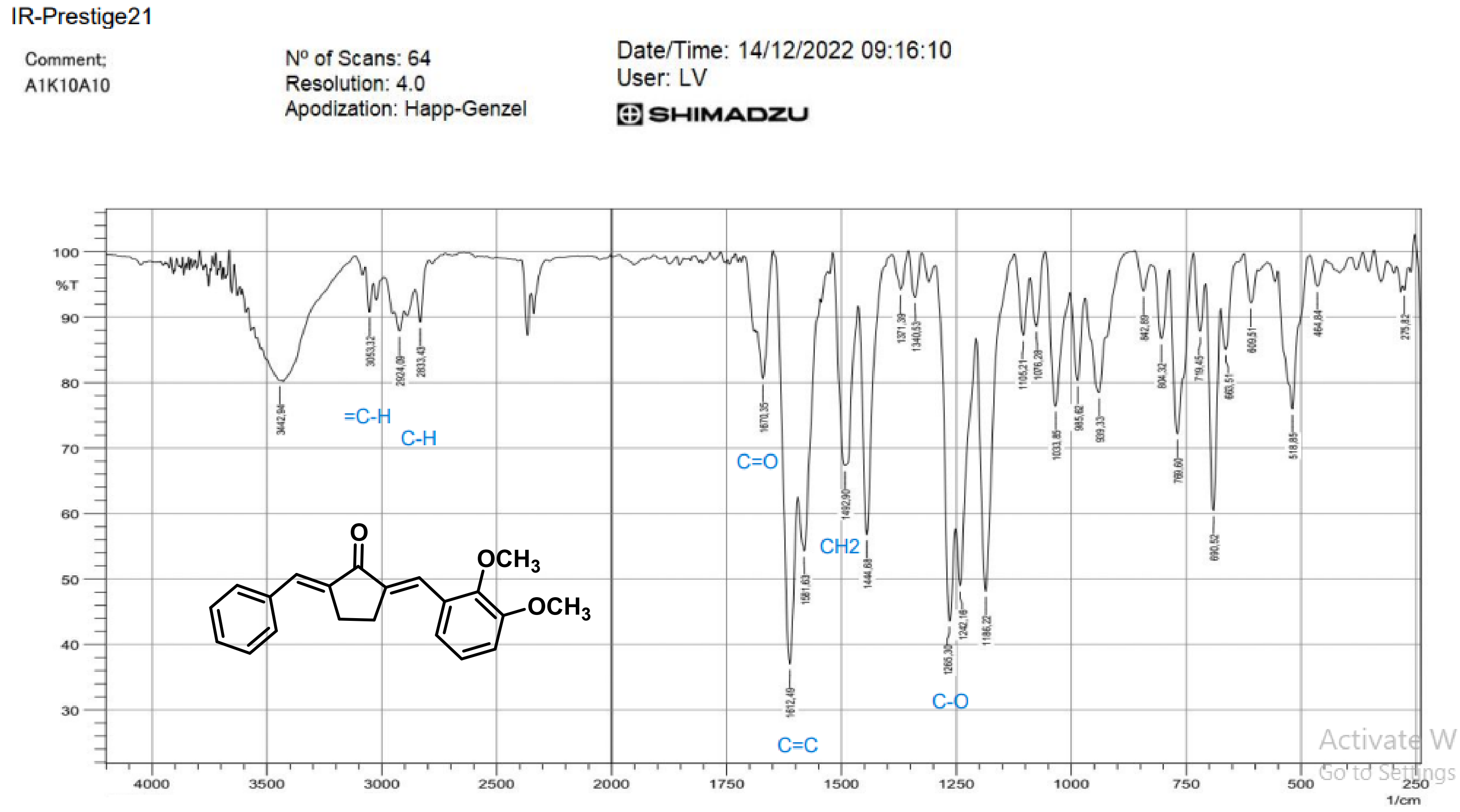

Fig. S6 IR spectrum of the compound **BDBC.**

checkCIF/PLATON report of BNTP.

Structure factors have been supplied for datablock(s) A1K7A11

THIS REPORT IS FOR GUIDANCE ONLY. IF USED AS PART OF A REVIEW PROCEDURE FOR PUBLICATION, IT SHOULD NOT REPLACE THE EXPERTISE OF AN EXPERIENCED CRYSTALLOGRAPHIC REFEREE.

No syntax errors found. CIF dictionary Interpreting this report

Datablock: BNTP

Bond precision:	C-C = 0.0021 A		Wavelength=0.71073
Cell:	a=7.5877(4)	b=7.2192(4)	c=28.0023(14)
	alpha=90	beta=90.507(1)	gamma=90
Temperature:	296 K		

	Calculated	Reported
Volume	1533.83(14)	1533.83(14)
Space group	P 21/n	P 21/n
Hall group	-P 2yn	-P 2yn
Moiety formula	C19 H15 N O4	C19 H15 N O4
Sum formula	C19 H15 N O4	C19 H15 N O4
Mr	321.32	321.32
Dx,g cm-3	1.391	1.391
Z	4	4
Mu (mm-1)	0.098	0.098
F000	672.0	672.0
F000’	672.35	
h,k,lmax	9,9,35	9,9,35
Nref	3262	3262
Tmin,Tmax	0.963,0.982	0.983,0.988
Tmin’	0.963	

Correction method= # Reported T Limits: Tmin=0.983 Tmax=0.988
AbsCorr = MULTI-SCAN

Data completeness= 1.000 Theta(max)= 26.729

R(reflections)= 0.0413(2850) wR2(reflections)= 0.1095(3262)

S = 1.029 Npar= 217

The following ALERTS were generated. Each ALERT has the format

test-name_ALERT_alert-type_alert-level.

Click on the hyperlinks for more details of the test.

Alert level G

```
PLAT978_ALERT_2_G Number C-C Bonds with Positive Residual Density.         15 Info
PLAT992_ALERT_5_G Repd & Actual _reflns_number_gt Values Differ by          2 Check
```

---

0 **ALERT level A** = Most likely a serious problem - resolve or explain

0 **ALERT level B** = A potentially serious problem, consider carefully

0 **ALERT level C** = Check. Ensure it is not caused by an omission or oversight

2 **ALERT level G** = General information/check it is not something unexpected

```
0 ALERT type 1 CIF construction/syntax error, inconsistent or missing data
1 ALERT type 2 Indicator that the structure model may be wrong or deficient
0 ALERT type 3 Indicator that the structure quality may be low
0 ALERT type 4 Improvement, methodology, query or suggestion
1 ALERT type 5 Informative message, check
```

It is advisable to attempt to resolve as many as possible of the alerts in all categories. Often the minor alerts point to easily fixed oversights, errors and omissions in your CIF or refinement strategy, so attention to these fine details can be worthwhile. In order to resolve some of the more serious problems it may be necessary to carry out additional measurements or structure refinements. However, the purpose of your study may justify the reported deviations and the more serious of these should normally be commented upon in the discussion or experimental section of a paper or in the "special_details" fields of the CIF. checkCIF was carefully designed to identify outliers and unusual parameters, but every test has its limitations and alerts that are not important in a particular case may appear. Conversely, the absence of alerts does not guarantee there are no aspects of the results needing attention. It is up to the individual to critically assess their own results and, if necessary, seek expert advice.

Publication of your CIF in IUCr journals

A basic structural check has been run on your CIF. These basic checks will be run on all CIFs submitted for publication in IUCr journals (*Acta Crystallographica*, *Journal of Applied Crystallography*, *Journal of Synchrotron Radiation*); however, if you intend to submit to *Acta Crystallographica Section C* or *E* or *IUCrData*, you should make sure that full publication checks are run on the final version of your CIF prior to submission.

Publication of your CIF in other journals

Please refer to the *Notes for Authors* of the relevant journal for any special instructions relating to CIF submission.

PLATON version of 19/02/2022; check.def file version of 19/02/2022

Datablock A1K7A11 - ellipsoid plot

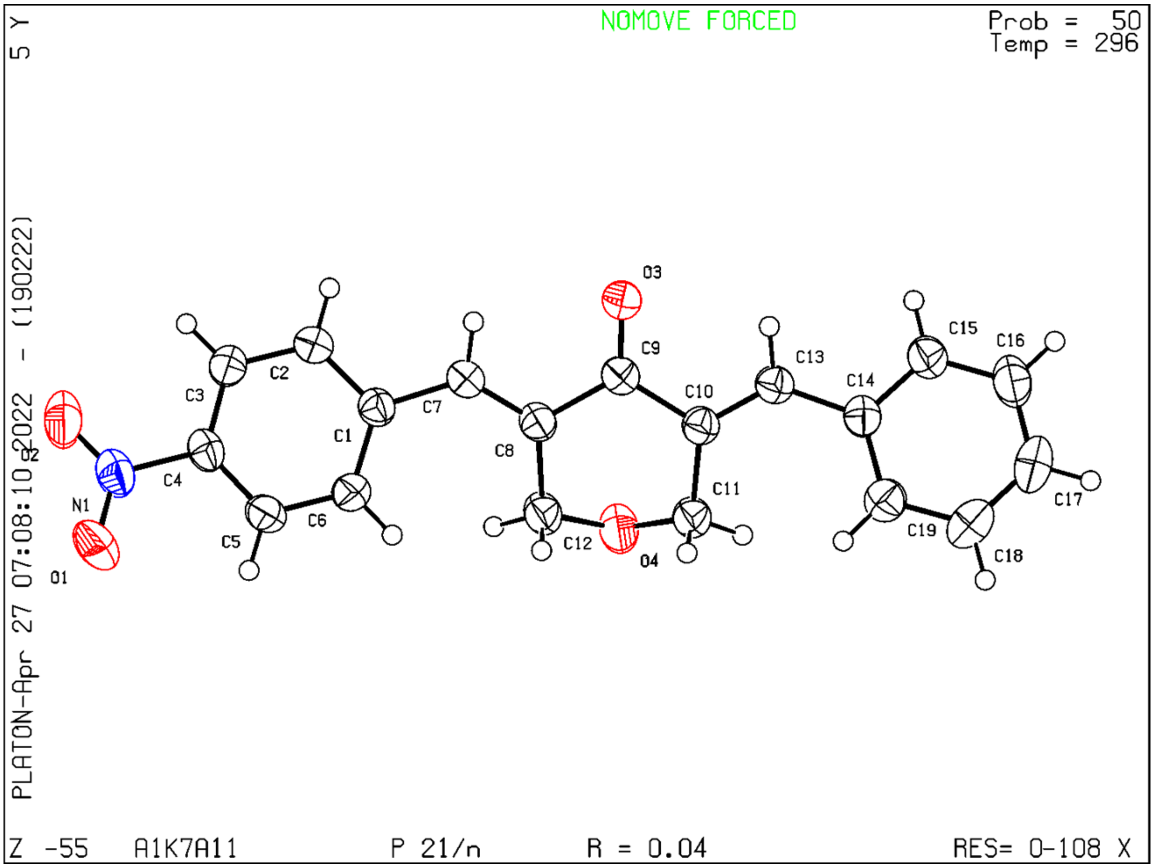

checkCIF/PLATON report of BDBC.

Structure factors have been supplied for datablock(s) A1K10A10

THIS REPORT IS FOR GUIDANCE ONLY. IF USED AS PART OF A REVIEW PROCEDURE FOR PUBLICATION, IT SHOULD NOT REPLACE THE EXPERTISE OF AN EXPERIENCED CRYSTALLOGRAPHIC REFEREE.

No syntax errors found. CIF dictionary Interpreting this report

Datablock: BDBC

Bond precision: C-C = 0.0034 A Wavelength=0.71073

Cell:	a=16.154(2)	b=6.9813(10)	c=28.997(4)
	alpha=90	beta=90	gamma=90
Temperature:	150 K		

	Calculated	Reported
Volume	3270.2(8)	3270.2(8)
Space group	P b c a	P b c a
Hall group	-P 2ac 2ab	-P 2ac 2ab
Moiety formula	C21 H20 O3	C21 H20 O3
Sum formula	C21 H20 O3	C21 H20 O3
Mr	320.37	320.37
Dx,g cm-3	1.301	1.301
Z	8	8
Mu (mm-1)	0.086	0.086
F000	1360.0	1360.0
F000'	1360.65	
h,k,lmax	19,8,34	19,8,34
Nref	2991	2992
Tmin,Tmax	0.969,0.983	0.983,0.988
Tmin'	0.969	

Correction method= # Reported T Limits: Tmin=0.983 Tmax=0.988
AbsCorr = MULTI-SCAN

Data completeness= 1.000 Theta(max)= 25.347

R(reflections)= 0.0547(2623) wR2(reflections)= 0.1161(2992)

S = 1.159 Npar= 219

The following ALERTS were generated. Each ALERT has the format

test-name_ALERT_alert-type_alert-level.

Click on the hyperlinks for more details of the test.

Alert level C

PLAT906_ALERT_3_C Large K Value in the Analysis of Variance ...... 7.164 Check

---

Alert level G

PLAT978_ALERT_2_G Number C-C Bonds with Positive Residual Density. 4 Info

---

0 **ALERT level A** = Most likely a serious problem - resolve or explain

0 **ALERT level B** = A potentially serious problem, consider carefully
1 **ALERT level C** = Check. Ensure it is not caused by an omission or oversight
1 **ALERT level G** = General information/check it is not something unexpected

0 ALERT type 1 CIF construction/syntax error, inconsistent or missing data
1 ALERT type 2 Indicator that the structure model may be wrong or deficient
1 ALERT type 3 Indicator that the structure quality may be low
0 ALERT type 4 Improvement, methodology, query or suggestion
0 ALERT type 5 Informative message, check

It is advisable to attempt to resolve as many as possible of the alerts in all categories. Often the minor alerts point to easily fixed oversights, errors and omissions in your CIF or refinement strategy, so attention to these fine details can be worthwhile. In order to resolve some of the more serious problems it may be necessary to carry out additional measurements or structure refinements. However, the purpose of your study may justify the reported deviations and the more serious of these should normally be commented upon in the discussion or experimental section of a paper or in the "special_details" fields of the CIF. checkCIF was carefully designed to identify outliers and unusual parameters, but every test has its limitations and alerts that are not important in a particular case may appear. Conversely, the absence of alerts does not guarantee there are no aspects of the results needing attention. It is up to the individual to critically assess their own results and, if necessary, seek expert advice.

Publication of your CIF in IUCr journals

A basic structural check has been run on your CIF. These basic checks will be run on all CIFs submitted for publication in IUCr journals (*Acta Crystallographica*, *Journal of Applied Crystallography*, *Journal of Synchrotron Radiation*); however, if you intend to submit to *Acta Crystallographica Section C* or *E* or *IUCrData*, you should make sure that full publication checks are run on the final version of your CIF prior to submission.

Publication of your CIF in other journals

Please refer to the *Notes for Authors* of the relevant journal for any special instructions relating to CIF submission.

PLATON version of 19/02/2022; check.def file version of 19/02/2022

Datablock A1K10A10 - ellipsoid plot

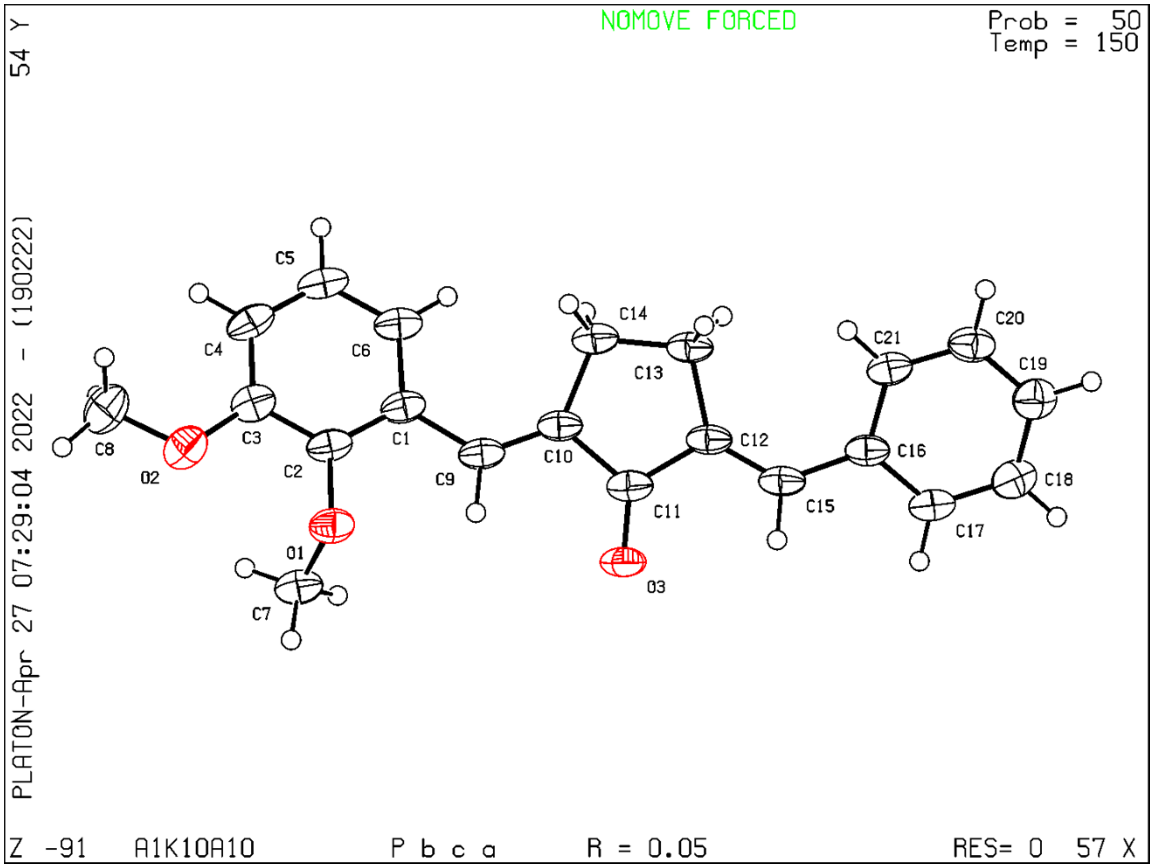

CIF of BNTP without HKL data

data_global

#==

PROCESSING SUMMARY (IUCr Office Use only)

_publ_contact_author

;

Muhammad Nawaz Tahir

University of Sargodha

Department of Physics

Sargodha

Pakistan

;

_publ_contact_author_phone '0092 48 92 30 914'

_publ_contact_author_fax '0092 48 32 22 121'

_publ_contact_author_email 'dmntahir_uos@yahoo.com'

_publ_requested_journal ''

_journal_date_recd_electronic ?

_journal_date_to_coeditor ?

_journal_date_from_coeditor ?

_journal_date_accepted ?

_journal_date_printers_first ?

_journal_date_printers_final ?

_journal_date_proofs_out ?

_journal_date_proofs_in ?

_journal_coeditor_name ?

_journal_coeditor_code ?

```
_journal_paper_category     ?
_journal_coeditor_notes
;
;

_journal_techeditor_code     ?
_iucr_compatibility_tag        ?
_journal_techeditor_notes
;
;

_journal_coden_ASTM             ?
_journal_name_full              ?
_journal_year                   ?
_journal_volume                 ?
_journal_issue                  ?
_journal_page_first             ?
_journal_page_last              ?

_journal_suppl_publ_number       ?
_journal_suppl_publ_pages        ?
#==============================================================

 loop_
_publ_author_name
```

_publ_author_address

'Muhammad Ashfaq'

;

Department of Physics

University of Sargodha

Sargodha

Pakistan

;

'Muhammad Nawaz Tahir'

;

Department of Physics

University of Sargodha

Sargodha

Pakistan

;

'Aleksey Kuznetsov'

;

Departamento deQuímica, Campus Santiago Vitacura

Universidad Tecnica Federico Santa María

Av. Santa María 6400 Vitacura

Chile

;

'Akbar Ali'

;

Department of Chemistry

University of Sargodha

Sargodha-40100

Pakistan

;

_audit_creation_date ?

_audit_creation_method ?

#==

TEXT

_publ_section_title

;

(3E,5E)-3-benzylidene-5-(4-nitrobenzylidene)dihydro-2H-pyran-4(3H)-one

;

_publ_section_abstract

;

;

_publ_section_comment

;

;

_publ_section_exptl_refinement

;

;

_publ_section_exptl_prep

;

;

_publ_section_related_literature

;

;

_publ_section_references

;

Bruker (2007). <i>SADABS</i>. Bruker AXS Inc., Madison, Wisconsin, USA.

Bruker (2007). <i>APEX2</i> and <i>SAINT</i>. Bruker AXS Inc.,

Madison, Wisconsin, USA.

Farrugia, L. J. (2012). <i>J. Appl. Cryst.</i> 45, 849--854.

Sheldrick, G. M. (2014). <i>Acta Cryst.</i> A70, C1437.

Sheldrick, G. M. (2015). <i>Acta Cryst.</i> C71, 3--8.

Spek, A. L. (2009). <i>Acta Cryst.</i> D65, 148--155.

;

_publ_section_figure_captions

;

;

_publ_section_acknowledgements

;

;

data_A1K7A11

_audit_creation_method 'SHELXL-2019/2'

_shelx_SHELXL_version_number '2019/2'

_chemical_name_common ?

_chemical_absolute_configuration '.'

_chemical_name_systematic

;

(3E,5E)-3-benzylidene-5-(4-nitrobenzylidene)dihydro-2H-pyran-4(3H)-one

;

_chemical_formula_moiety 'C19 H15 N O4'

_chemical_formula_sum 'C19 H15 N O4'

_chemical_formula_iupac 'C19 H15 N O4'

_chemical_formula_weight 321.32

loop_

_atom_type_symbol

_atom_type_description

_atom_type_scat_dispersion_real

_atom_type_scat_dispersion_imag

_atom_type_scat_source

'C' 'C' 0.0033 0.0016

'International Tables Vol C Tables 4.2.6.8 and 6.1.1.4'

'H' 'H' 0.0000 0.0000

'International Tables Vol C Tables 4.2.6.8 and 6.1.1.4'

'N' 'N' 0.0061 0.0033

'International Tables Vol C Tables 4.2.6.8 and 6.1.1.4'

'O' 'O' 0.0106 0.0060

'International Tables Vol C Tables 4.2.6.8 and 6.1.1.4'

_space_group_crystal_system monoclinic

_space_group_IT_number 14

_space_group_name_H-M_alt 'P 21/n'

_space_group_name_Hall '-P 2yn'

_shelx_space_group_comment

;

The symmetry employed for this shelxl refinement is uniquely defined

by the following loop, which should always be used as a source of

symmetry information in preference to the above space-group names.

They are only intended as comments.

;

loop_

 _space_group_symop_operation_xyz

 'x, y, z'

 '-x+1/2, y+1/2, -z+1/2'

 '-x, -y, -z'

 'x-1/2, -y-1/2, z-1/2'

_cell_length_a 7.5877(4)

_cell_length_b 7.2192(4)

_cell_length_c 28.0023(14)

_cell_angle_alpha 90

_cell_angle_beta 90.5070(10)

_cell_angle_gamma 90

_cell_volume 1533.83(14)

_cell_formula_units_Z 4

_cell_measurement_reflns_used 2850

_cell_measurement_theta_min 1.454

_cell_measurement_theta_max 26.729

_cell_measurement_temperature 296(2)

_exptl_crystal_description needle

_exptl_crystal_colour 'white'

_exptl_crystal_size_max 0.381

_exptl_crystal_size_mid 0.375

_exptl_crystal_size_min 0.186

_exptl_crystal_density_diffrn 1.391

_exptl_crystal_density_meas ?

_exptl_crystal_density_method 'not measured'

_exptl_crystal_F_000 672

_exptl_absorpt_coefficient_mu 0.098

_exptl_absorpt_correction_type multi-scan

_exptl_absorpt_process_details '(SADABS; Bruker, 2007)'

_exptl_absorpt_correction_T_min 0.983

_exptl_absorpt_correction_T_max 0.988

_exptl_special_details

;

;

_diffrn_ambient_temperature 296(2)

_diffrn_radiation_type MoK\a

_diffrn_radiation_wavelength 0.71073

_diffrn_radiation_source 'fine-focus sealed tube'

_diffrn_radiation_monochromator graphite

_diffrn_measurement_device_type 'Bruker Kappa APEXII CCD'

_diffrn_measurement_method \w

_diffrn_detector_area_resol_mean 7.901

_diffrn_reflns_number 24896

_diffrn_reflns_av_unetI/netI 0.0107

_diffrn_reflns_av_R_equivalents 0.0194

_diffrn_reflns_limit_h_min -9

_diffrn_reflns_limit_h_max 9

_diffrn_reflns_limit_k_min -9

_diffrn_reflns_limit_k_max 9

_diffrn_reflns_limit_l_min -35

_diffrn_reflns_limit_l_max 35

_diffrn_reflns_theta_min 1.454

_diffrn_reflns_theta_max 26.729

_diffrn_reflns_theta_full 25.242

_diffrn_measured_fraction_theta_max 1.000

_diffrn_measured_fraction_theta_full 1.000

_diffrn_reflns_Laue_measured_fraction_max 1.000

_diffrn_reflns_Laue_measured_fraction_full 1.000

_diffrn_reflns_point_group_measured_fraction_max 1.000

_diffrn_reflns_point_group_measured_fraction_full 1.000

_diffrn_standards_number 0

_diffrn_standards_interval_count ?

_diffrn_standards_interval_time ?

_diffrn_standards_decay_% ?

_refine_special_details

;

Refinement of <i>F</i>^2^ against ALL reflections. The weighted <i>R</i>-factor

<i>wR</i> and goodness of fit <i>S</i> are based on <i>F</i>^2^, conventional

<i>R</i>-factors <i>R</i> are based on <i>F</i>, with <i>F</i> set to zero for

negative <i>F</i>^2^. The threshold expression of <i>F</i>^2^ >

\s(<i>F</i>^2^) is used only for calculating <i>R</i>-factors(gt) <i>etc</i>.

and is not relevant to the choice of reflections for refinement.

<i>R</i>-factors based on <i>F</i>^2^ are statistically about twice as large

as those based on <i>F</i>, and <i>R</i>- factors based on ALL data will be

even larger.

;

_reflns_number_total 3262

_reflns_number_gt 2850

_reflns_threshold_expression I>2\s(I)

_refine_ls_structure_factor_coef Fsqd

_refine_ls_matrix_type full

_refine_ls_R_factor_all 0.0474

_refine_ls_R_factor_gt 0.0413

_refine_ls_wR_factor_ref 0.1095

_refine_ls_wR_factor_gt 0.1044

_refine_ls_goodness_of_fit_ref 1.029

_refine_ls_restrained_S_all 1.029

_refine_ls_number_reflns 3262

_refine_ls_number_parameters 217

_refine_ls_number_restraints 0

_refine_ls_extinction_method none

_refine_ls_extinction_coef .

_refine_ls_hydrogen_treatment constr

_refine_ls_weighting_scheme calc

_refine_ls_weighting_details

'w=1/[\s^2^(Fo^2^)+(0.0472P)^2^+0.5545P] where P=(Fo^2^+2Fc^2^)/3'

_atom_sites_solution_hydrogens geom

_atom_sites_solution_primary direct

_atom_sites_solution_secondary difmap

_refine_ls_shift/su_max 0.000

_refine_ls_shift/su_mean 0.000

_computing_data_collection 'APEX2 (Bruker, 2007)'

_computing_cell_refinement 'SAINT (Bruker, 2007)'

_computing_data_reduction 'SAINT (Bruker, 2007)'

_computing_structure_solution 'SHELXT-2015 (Sheldrick, 2015)'

_computing_structure_refinement 'SHELXL-2019/2 (Sheldrick, 2019)'

_computing_molecular_graphics

'ORTEP-3 for Windows (Farrugia, 2012) and PLATON (Spek, 2009)'

_computing_publication_material

;

WinGX (Farrugia, 2012) and PLATON (Spek, 2009)

;

```
loop_
 _atom_site_label
 _atom_site_type_symbol
 _atom_site_fract_x
 _atom_site_fract_y
 _atom_site_fract_z
 _atom_site_U_iso_or_equiv
 _atom_site_adp_type
 _atom_site_occupancy
 _atom_site_site_symmetry_order
 _atom_site_calc_flag
 _atom_site_refinement_flags_posn
 _atom_site_refinement_flags_adp
 _atom_site_refinement_flags_occupancy
 _atom_site_disorder_assembly
 _atom_site_disorder_group
O1 O -0.32508(16) 0.6976(2) -0.15387(5) 0.0703(4) Uani 1 1 d . . . . .
O2 O -0.16115(18) 0.5255(2) -0.19751(5) 0.0798(4) Uani 1 1 d . . . . .
O3 O 0.71000(14) 0.7812(2) 0.01037(4) 0.0708(4) Uani 1 1 d . . . . .
O4 O 0.30539(14) 0.68285(17) 0.09411(4) 0.0539(3) Uani 1 1 d . . . . .
N1 N -0.18561(17) 0.6204(2) -0.16233(5) 0.0513(3) Uani 1 1 d . . . . .
C1 C 0.23546(17) 0.69275(19) -0.06442(5) 0.0368(3) Uani 1 1 d . . . . .
C2 C 0.26412(18) 0.6197(2) -0.10970(5) 0.0411(3) Uani 1 1 d . . . . .
H2 H 0.378162 0.588637 -0.118585 0.049 Uiso 1 1 calc R U . . .
C3 C 0.12725(19) 0.5924(2) -0.14169(5) 0.0423(3) Uani 1 1 d . . . . .
```

H3 H 0.147391 0.541166 -0.171616 0.051 Uiso 1 1 calc R U . . .

C4 C -0.03952(18) 0.64298(19) -0.12818(5) 0.0393(3) Uani 1 1 d . . . . .

C5 C -0.07372(19) 0.7177(2) -0.08424(5) 0.0454(3) Uani 1 1 d . . . . .

H5 H -0.187833 0.751522 -0.076101 0.054 Uiso 1 1 calc R U . . .

C6 C 0.06367(19) 0.7418(2) -0.05224(5) 0.0448(3) Uani 1 1 d . . . . .

H6 H 0.041600 0.791403 -0.022231 0.054 Uiso 1 1 calc R U . . .

C7 C 0.38933(18) 0.7217(2) -0.03336(5) 0.0403(3) Uani 1 1 d . . . . .

H7 H 0.496814 0.723396 -0.049062 0.048 Uiso 1 1 calc R U . . .

C8 C 0.40033(17) 0.74602(19) 0.01377(5) 0.0382(3) Uani 1 1 d . . . . .

C9 C 0.57861(18) 0.7828(2) 0.03507(5) 0.0420(3) Uani 1 1 d . . . . .

C10 C 0.58892(18) 0.82720(19) 0.08686(5) 0.0372(3) Uani 1 1 d . . . . .

C11 C 0.41995(19) 0.8177(2) 0.11433(5) 0.0461(4) Uani 1 1 d . . . . .

H11A H 0.445436 0.786103 0.147338 0.055 Uiso 1 1 calc R U . . .

H11B H 0.362768 0.937884 0.113781 0.055 Uiso 1 1 calc R U . . .

C12 C 0.24838(19) 0.7373(3) 0.04774(5) 0.0490(4) Uani 1 1 d . . . . .

H12A H 0.192562 0.857981 0.049484 0.059 Uiso 1 1 calc R U . . .

H12B H 0.161719 0.649494 0.035877 0.059 Uiso 1 1 calc R U . . .

C13 C 0.74777(18) 0.86477(19) 0.10560(5) 0.0392(3) Uani 1 1 d . . . . .

H13 H 0.840759 0.853469 0.084384 0.047 Uiso 1 1 calc R U . . .

C14 C 0.79921(18) 0.92056(19) 0.15385(5) 0.0382(3) Uani 1 1 d . . . . .

C15 C 0.9736(2) 0.8908(2) 0.16801(5) 0.0474(4) Uani 1 1 d . . . . .

H15 H 1.050513 0.832369 0.147173 0.057 Uiso 1 1 calc R U . . .

C16 C 1.0341(2) 0.9463(3) 0.21224(6) 0.0588(4) Uani 1 1 d . . . . .

H16 H 1.150418 0.923623 0.221233 0.071 Uiso 1 1 calc R U . . .

C17 C 0.9228(3) 1.0350(3) 0.24302(6) 0.0609(5) Uani 1 1 d . . . . .

H17 H 0.963743 1.073189 0.272828 0.073 Uiso 1 1 calc R U . . .

C18 C 0.7506(3) 1.0674(2) 0.22986(6) 0.0556(4) Uani 1 1 d . . . . .

H18 H 0.675650 1.127991 0.250797 0.067 Uiso 1 1 calc R U . . .

C19 C 0.6881(2) 1.0105(2) 0.18572(5) 0.0453(3) Uani 1 1 d . . . . .

H19 H 0.571210 1.032422 0.177244 0.054 Uiso 1 1 calc R U . . .

loop_

_atom_site_aniso_label

_atom_site_aniso_U_11

_atom_site_aniso_U_22

_atom_site_aniso_U_33

_atom_site_aniso_U_23

_atom_site_aniso_U_13

_atom_site_aniso_U_12

O1 0.0410(6) 0.0954(10) 0.0742(9) 0.0037(7) -0.0155(6) 0.0116(7)

O2 0.0713(9) 0.1046(11) 0.0630(8) -0.0256(8) -0.0292(7) 0.0127(8)

O3 0.0358(6) 0.1329(13) 0.0438(6) -0.0222(7) 0.0033(5) -0.0064(7)

O4 0.0480(6) 0.0744(8) 0.0393(5) 0.0128(5) -0.0040(4) -0.0218(5)

N1 0.0443(7) 0.0599(8) 0.0495(7) 0.0057(6) -0.0128(6) -0.0007(6)

C1 0.0348(7) 0.0395(7) 0.0362(7) 0.0028(5) -0.0015(5) -0.0006(5)

C2 0.0346(7) 0.0474(8) 0.0415(7) -0.0025(6) 0.0010(6) 0.0031(6)

C3 0.0435(8) 0.0460(8) 0.0374(7) -0.0040(6) -0.0028(6) 0.0018(6)

C4 0.0364(7) 0.0413(7) 0.0402(7) 0.0066(6) -0.0071(6) -0.0027(6)

C5 0.0328(7) 0.0602(9) 0.0432(8) 0.0035(7) 0.0011(6) 0.0042(6)

C6 0.0392(8) 0.0602(9) 0.0350(7) -0.0012(6) 0.0008(6) 0.0060(7)

C7 0.0330(7) 0.0472(8) 0.0405(7) -0.0019(6) -0.0011(5) 0.0015(6)

C8 0.0343(7) 0.0413(7) 0.0389(7) 0.0013(6) -0.0024(5) -0.0007(6)

C9 0.0346(7) 0.0523(8) 0.0391(7) -0.0011(6) -0.0002(6) -0.0007(6)

C10 0.0363(7) 0.0404(7) 0.0350(7) 0.0020(5) -0.0005(5) 0.0001(6)

C11 0.0391(8) 0.0626(9) 0.0366(7) 0.0019(7) 0.0001(6) -0.0064(7)

C12 0.0371(8) 0.0708(10) 0.0390(7) 0.0060(7) -0.0022(6) -0.0097(7)

C13 0.0360(7) 0.0431(7) 0.0383(7) 0.0008(6) 0.0012(5) -0.0008(6)

C14 0.0398(7) 0.0381(7) 0.0367(7) 0.0036(5) -0.0020(5) -0.0056(6)

C15 0.0401(8) 0.0566(9) 0.0455(8) -0.0005(7) -0.0022(6) -0.0050(7)

C16 0.0511(9) 0.0729(11) 0.0522(9) 0.0009(8) -0.0146(7) -0.0090(8)

C17 0.0793(12) 0.0625(11) 0.0406(8) -0.0035(8) -0.0143(8) -0.0098(9)

C18 0.0780(12) 0.0478(9) 0.0412(8) -0.0021(7) 0.0041(8) 0.0031(8)

C19 0.0488(8) 0.0432(8) 0.0439(8) 0.0018(6) -0.0010(6) 0.0032(6)

_geom_special_details

;

All esds (except the esd in the dihedral angle between two l.s. planes)

are estimated using the full covariance matrix. The cell esds are taken

into account individually in the estimation of esds in distances, angles

and torsion angles; correlations between esds in cell parameters are only

used when they are defined by crystal symmetry. An approximate (isotropic)

treatment of cell esds is used for estimating esds involving l.s. planes.

;

loop_

_geom_bond_atom_site_label_1

_geom_bond_atom_site_label_2

_geom_bond_distance

_geom_bond_site_symmetry_2

_geom_bond_publ_flag

O1 N1 1.2209(18) . ?

O2 N1 1.2156(18) . ?

O3 C9 1.2185(18) . ?

O4 C11 1.4197(18) . ?

O4 C12 1.4204(17) . ?

N1 C4 1.4666(18) . ?

C1 C2 1.3918(19) . ?

C1 C6 1.3960(19) . ?

C1 C7 1.4647(18) . ?

C2 C3 1.3798(19) . ?

C2 H2 0.9300 . ?

C3 C4 1.373(2) . ?

C3 H3 0.9300 . ?

C4 C5 1.370(2) . ?

C5 C6 1.380(2) . ?

C5 H5 0.9300 . ?

C6 H6 0.9300 . ?

C7 C8 1.3334(19) . ?

C7 H7 0.9300 . ?

C8 C9 1.4974(19) . ?

C8 C12 1.502(2) . ?

C9 C10 1.4863(19) . ?

C10 C13 1.3382(19) . ?

C10 C11 1.5025(19) . ?

C11 H11A 0.9700 . ?

C11 H11B 0.9700 . ?

C12 H12A 0.9700 . ?

C12 H12B 0.9700 . ?

C13 C14 1.4597(19) . ?

C13 H13 0.9300 . ?

C14 C19 1.393(2) . ?

C14 C15 1.395(2) . ?

C15 C16 1.377(2) . ?

C15 H15 0.9300 . ?

C16 C17 1.371(3) . ?

C16 H16 0.9300 . ?

C17 C18 1.374(3) . ?

C17 H17 0.9300 . ?

C18 C19 1.382(2) . ?

C18 H18 0.9300 . ?

C19 H19 0.9300 . ?

loop_

_geom_angle_atom_site_label_1

_geom_angle_atom_site_label_2

_geom_angle_atom_site_label_3

_geom_angle

_geom_angle_site_symmetry_1

_geom_angle_site_symmetry_3

_geom_angle_publ_flag

C11 O4 C12 110.80(12) . . ?

O2 N1 O1 123.58(14) . . ?

O2 N1 C4 118.10(13) . . ?

O1 N1 C4 118.31(14) . . ?

C2 C1 C6 118.19(12) . . ?

C2 C1 C7 117.70(12) . . ?

C6 C1 C7 124.04(13) . . ?

C3 C2 C1 121.52(13) . . ?

C3 C2 H2 119.2 . . ?

C1 C2 H2 119.2 . . ?

C4 C3 C2 118.25(13) . . ?

C4 C3 H3 120.9 . . ?

C2 C3 H3 120.9 . . ?

C5 C4 C3 122.28(13) . . ?

C5 C4 N1 118.76(13) . . ?

C3 C4 N1 118.94(13) . . ?

C4 C5 C6 119.01(13) . . ?

C4 C5 H5 120.5 . . ?

C6 C5 H5 120.5 . . ?

C5 C6 C1 120.74(13) . . ?

C5 C6 H6 119.6 . . ?

C1 C6 H6 119.6 . . ?

C8 C7 C1 130.48(13) . . ?

C8 C7 H7 114.8 . . ?

C1 C7 H7 114.8 . . ?

C7 C8 C9 117.78(13) . . ?

C7 C8 C12 125.42(13) . . ?

C9 C8 C12 116.80(12) . . ?

O3 C9 C10 121.31(13) . . ?

O3 C9 C8 120.85(13) . . ?

C10 C9 C8 117.80(12) . . ?

C13 C10 C9 117.76(12) . . ?

C13 C10 C11 125.32(13) . . ?

C9 C10 C11 116.86(12) . . ?

O4 C11 C10 110.45(12) . . ?

O4 C11 H11A 109.6 . . ?

C10 C11 H11A 109.6 . . ?

O4 C11 H11B 109.6 . . ?

C10 C11 H11B 109.6 . . ?

H11A C11 H11B 108.1 . . ?

O4 C12 C8 111.17(12) . . ?

O4 C12 H12A 109.4 . . ?

C8 C12 H12A 109.4 . . ?

O4 C12 H12B 109.4 . . ?

C8 C12 H12B 109.4 . . ?

H12A C12 H12B 108.0 . . ?

C10 C13 C14 130.66(13) . . ?

C10 C13 H13 114.7 . . ?

C14 C13 H13 114.7 . . ?

C19 C14 C15 117.87(13) . . ?

C19 C14 C13 124.27(13) . . ?

C15 C14 C13 117.77(13) . . ?

C16 C15 C14 121.26(15) . . ?

C16 C15 H15 119.4 . . ?

C14 C15 H15 119.4 . . ?

C17 C16 C15 119.93(16) . . ?

C17 C16 H16 120.0 . . ?

C15 C16 H16 120.0 . . ?

C16 C17 C18 120.04(15) . . ?

C16 C17 H17 120.0 . . ?

C18 C17 H17 120.0 . . ?

C17 C18 C19 120.47(16) . . ?

C17 C18 H18 119.8 . . ?

C19 C18 H18 119.8 . . ?

C18 C19 C14 120.43(15) . . ?

C18 C19 H19 119.8 . . ?

C14 C19 H19 119.8 . . ?

loop_

_geom_torsion_atom_site_label_1

_geom_torsion_atom_site_label_2

_geom_torsion_atom_site_label_3

_geom_torsion_atom_site_label_4

_geom_torsion

_geom_torsion_site_symmetry_1

_geom_torsion_site_symmetry_2

_geom_torsion_site_symmetry_3

_geom_torsion_site_symmetry_4

_geom_torsion_publ_flag

C6 C1 C2 C3 -1.3(2) . . . . ?

C7 C1 C2 C3 -178.32(13) . . . . ?

C1 C2 C3 C4 1.4(2) . . . . ?

C2 C3 C4 C5 -0.5(2) . . . . ?

C2 C3 C4 N1 178.06(13) . . . . ?

O2 N1 C4 C5 -167.28(16) . . . . ?

O1 N1 C4 C5 12.6(2) . . . . ?

O2 N1 C4 C3 14.1(2) . . . . ?

O1 N1 C4 C3 -165.99(15) . . . . ?

C3 C4 C5 C6 -0.5(2) . . . . ?

N1 C4 C5 C6 -179.01(14) . . . . ?

C4 C5 C6 C1 0.6(2) . . . . ?

C2 C1 C6 C5 0.3(2) . . . . ?

C7 C1 C6 C5 177.14(14) . . . . ?

C2 C1 C7 C8 -162.09(15) . . . . ?

C6 C1 C7 C8 21.0(2) . . . . ?

C1 C7 C8 C9 -176.74(14) . . . . ?

C1 C7 C8 C12 4.1(3) . . . . ?

C7 C8 C9 O3 -3.6(2) . . . . ?

C12 C8 C9 O3 175.64(16) . . . . ?

C7 C8 C9 C10 174.46(13) . . . . ?

C12 C8 C9 C10 -6.3(2) . . . . ?

O3 C9 C10 C13 -0.2(2) . . . . ?

C8 C9 C10 C13 -178.32(13) . . . . ?

O3 C9 C10 C11 -177.59(16) . . . . ?

C8 C9 C10 C11 4.3(2) . . . . ?

C12 O4 C11 C10 -66.43(16) . . . . ?

C13 C10 C11 O4 -146.79(14) . . . . ?

C9 C10 C11 O4 30.34(18) . . . . ?

C11 O4 C12 C8 64.52(17) . . . . ?

C7 C8 C12 O4 152.65(15) . . . . ?

C9 C8 C12 O4 -26.6(2) . . . . ?

C9 C10 C13 C14 176.86(14) . . . . ?

C11 C10 C13 C14 -6.0(3) . . . . ?

C10 C13 C14 C19 -24.8(2) . . . . ?

C10 C13 C14 C15 158.80(15) . . . . ?

C19 C14 C15 C16 0.8(2) . . . . ?

C13 C14 C15 C16 177.39(15) . . . . ?

C14 C15 C16 C17 -0.9(3) . . . . ?

C15 C16 C17 C18 0.4(3) . . . . ?

C16 C17 C18 C19 0.2(3) . . . . ?

C17 C18 C19 C14 -0.4(2) . . . . ?

C15 C14 C19 C18 -0.1(2) . . . . ?

C13 C14 C19 C18 -176.47(14) . . . . ?

loop_

_geom_hbond_atom_site_label_D

_geom_hbond_atom_site_label_H

_geom_hbond_atom_site_label_A

_geom_hbond_distance_DH

_geom_hbond_distance_HA

_geom_hbond_distance_DA

_geom_hbond_angle_DHA

_geom_hbond_site_symmetry_A

_geom_hbond_publ_flag

C5 H5 O3 0.93 2.56 3.1620(18) 123.0 1_455 yes

C2 H2 O1 0.93 2.59 3.4102(19) 147.5 1_655 yes

_refine_diff_density_max 0.189

_refine_diff_density_min -0.189

_refine_diff_density_rms 0.033

CIF of BDBC without HKL data

data_global

#==

PROCESSING SUMMARY (IUCr Office Use only)

_publ_contact_author

;

Muhammad Nawaz Tahir

University of Sargodha

Department of Physics

Sargodha

Pakistan

;

_publ_contact_author_phone '0092 48 92 30 914'

_publ_contact_author_fax '0092 48 32 22 121'

_publ_contact_author_email 'dmntahir_uos@yahoo.com'

_publ_requested_journal ''

_journal_date_recd_electronic ?

_journal_date_to_coeditor ?

_journal_date_from_coeditor ?

_journal_date_accepted ?

_journal_date_printers_first ?

_journal_date_printers_final ?

_journal_date_proofs_out ?

_journal_date_proofs_in ?

_journal_coeditor_name ?

_journal_coeditor_code ?

_journal_paper_category ?

_journal_coeditor_notes

;

;

_journal_techeditor_code ?

_iucr_compatibility_tag ?

_journal_techeditor_notes

;

;

_journal_coden_ASTM ?

_journal_name_full ?

_journal_year ?

_journal_volume ?

_journal_issue ?

_journal_page_first ?

_journal_page_last ?

_journal_suppl_publ_number ?

_journal_suppl_publ_pages ?

#==

loop_

_publ_author_name

_publ_author_address

'Muhammad Ashfaq'

;

Department of Physics

University of Sargodha

Sargodha

Pakistan

;

'Muhammad Nawaz Tahir'

;

Department of Physics

University of Sargodha

Sargodha

Pakistan

;

'Aleksey Kuznetsov'

;

Departamento deQuímica, Campus Santiago Vitacura

Universidad Tecnica Federico Santa María

Av. Santa María 6400 Vitacura

Chile
;

'Akbar Ali'
;
Department of Chemistry
University of Sargodha
Sargodha-40100
Pakistan
;

_audit_creation_date ?

_audit_creation_method ?

#==

TEXT

_publ_section_title
;
(2E,5E)-2-benzylidene-5-(2,3-dimethoxybenzylidene)cyclopentanone
;

_publ_section_abstract
;

;

_publ_section_comment

;

;

_publ_section_exptl_refinement

;

;

_publ_section_exptl_prep

;

;

_publ_section_related_literature

;

;

_publ_section_references

;

Bruker (2007). <i>SADABS</i>. Bruker AXS Inc., Madison, Wisconsin, USA.

Bruker (2007). <i>APEX2</i> and <i>SAINT</i>. Bruker AXS Inc.,

Madison, Wisconsin, USA.

Farrugia, L. J. (2012). <i>J. Appl. Cryst.</i> 45, 849--854.

Sheldrick, G. M. (2014). <i>Acta Cryst.</i> A70, C1437.

Sheldrick, G. M. (2015). <i>Acta Cryst.</i> C71, 3--8.

Spek, A. L. (2009). <i>Acta Cryst.</i> D65, 148--155.

;

_publ_section_figure_captions

;

;

_publ_section_acknowledgements

;

;

data_A1K10A10

_audit_creation_method 'SHELXL-2019/2'

_shelx_SHELXL_version_number '2019/2'

_chemical_name_common ?

_chemical_absolute_configuration '.'

_chemical_name_systematic

;

(2E,5E)-2-benzylidene-5-(2,3-dimethoxybenzylidene)cyclopentanone

;

_chemical_formula_moiety 'C21 H20 O3'

_chemical_formula_sum 'C21 H20 O3'

_chemical_formula_iupac 'C21 H20 O3'

_chemical_formula_weight 320.37

loop_

_atom_type_symbol

_atom_type_description

_atom_type_scat_dispersion_real

_atom_type_scat_dispersion_imag

_atom_type_scat_source

'C' 'C' 0.0033 0.0016

'International Tables Vol C Tables 4.2.6.8 and 6.1.1.4'

'H' 'H' 0.0000 0.0000

'International Tables Vol C Tables 4.2.6.8 and 6.1.1.4'

'O' 'O' 0.0106 0.0060

'International Tables Vol C Tables 4.2.6.8 and 6.1.1.4'

_space_group_crystal_system orthorhombic

_space_group_IT_number 61

_space_group_name_H-M_alt 'P b c a'

_space_group_name_Hall '-P 2ac 2ab'

_shelx_space_group_comment

;

The symmetry employed for this shelxl refinement is uniquely defined

by the following loop, which should always be used as a source of

symmetry information in preference to the above space-group names.

They are only intended as comments.

;

loop_

_space_group_symop_operation_xyz

'x, y, z'

'x+1/2, -y+1/2, -z'

'-x, y+1/2, -z+1/2'

'-x+1/2, -y, z+1/2'

'-x, -y, -z'

'-x-1/2, y-1/2, z'

'x, -y-1/2, z-1/2'

'x-1/2, y, -z-1/2'

_cell_length_a 16.154(2)

_cell_length_b 6.9813(10)

_cell_length_c 28.997(4)

_cell_angle_alpha 90

_cell_angle_beta 90

_cell_angle_gamma 90

_cell_volume 3270.2(8)

_cell_formula_units_Z 8

_cell_measurement_reflns_used 2623

_cell_measurement_theta_min 1.404

_cell_measurement_theta_max 25.347

_cell_measurement_temperature 150(2)

_exptl_crystal_description needle

_exptl_crystal_colour 'white'

_exptl_crystal_size_max 0.361

_exptl_crystal_size_mid 0.345

_exptl_crystal_size_min 0.194

_exptl_crystal_density_diffrn 1.301

_exptl_crystal_density_meas ?

_exptl_crystal_density_method 'not measured'

_exptl_crystal_F_000 1360

_exptl_absorpt_coefficient_mu 0.086

_exptl_absorpt_correction_type multi-scan

_exptl_absorpt_process_details '(SADABS; Bruker, 2007)'

_exptl_absorpt_correction_T_min 0.983

_exptl_absorpt_correction_T_max 0.988

_exptl_special_details

;

;

_diffrn_ambient_temperature 150(2)

_diffrn_radiation_type MoK\a

_diffrn_radiation_wavelength 0.71073

_diffrn_radiation_source 'fine-focus sealed tube'

_diffrn_radiation_monochromator graphite

_diffrn_measurement_device_type 'Bruker Kappa APEXII CCD'

_diffrn_measurement_method \w

_diffrn_detector_area_resol_mean 8.301

_diffrn_reflns_number 57033

_diffrn_reflns_av_unetI/netI 0.0108

_diffrn_reflns_av_R_equivalents 0.0258

_diffrn_reflns_limit_h_min -19

_diffrn_reflns_limit_h_max 19

_diffrn_reflns_limit_k_min -8

_diffrn_reflns_limit_k_max 5

_diffrn_reflns_limit_l_min -34

_diffrn_reflns_limit_l_max 34

_diffrn_reflns_theta_min 1.404

_diffrn_reflns_theta_max 25.347

_diffrn_reflns_theta_full 25.242

_diffrn_measured_fraction_theta_max 1.000

_diffrn_measured_fraction_theta_full 1.000

_diffrn_reflns_Laue_measured_fraction_max 1.000

_diffrn_reflns_Laue_measured_fraction_full 1.000

_diffrn_reflns_point_group_measured_fraction_max 1.000

_diffrn_reflns_point_group_measured_fraction_full 1.000

_diffrn_standards_number 0

_diffrn_standards_interval_count ?

_diffrn_standards_interval_time ?

_diffrn_standards_decay_% ?

_refine_special_details

;

Refinement of <i>F</i>^2^ against ALL reflections. The weighted <i>R</i>-factor

<i>wR</i> and goodness of fit <i>S</i> are based on <i>F</i>^2^, conventional

<i>R</i>-factors <i>R</i> are based on <i>F</i>, with <i>F</i> set to zero for

negative <i>F</i>^2^. The threshold expression of <i>F</i>^2^ >

\s(<i>F</i>^2^) is used only for calculating <i>R</i>-factors(gt) <i>etc</i>.

and is not relevant to the choice of reflections for refinement.

<i>R</i>-factors based on <i>F</i>^2^ are statistically about twice as large

as those based on <i>F</i>, and <i>R</i>- factors based on ALL data will be

even larger.

;

_reflns_number_total 2992

_reflns_number_gt 2623

_reflns_threshold_expression I>2\s(I)

_refine_ls_structure_factor_coef Fsqd

_refine_ls_matrix_type full

_refine_ls_R_factor_all 0.0623

_refine_ls_R_factor_gt 0.0547

_refine_ls_wR_factor_ref 0.1161

_refine_ls_wR_factor_gt 0.1123

_refine_ls_goodness_of_fit_ref 1.159

_refine_ls_restrained_S_all 1.159

_refine_ls_number_reflns 2992

_refine_ls_number_parameters 219

_refine_ls_number_restraints 0

_refine_ls_extinction_method none

_refine_ls_extinction_coef .

_refine_ls_hydrogen_treatment constr

_refine_ls_weighting_scheme calc

_refine_ls_weighting_details

'w=1/[\s^2^(Fo^2^)+(0.0146P)^2^+4.2071P] where P=(Fo^2^+2Fc^2^)/3'

_atom_sites_solution_hydrogens geom

_atom_sites_solution_primary direct

_atom_sites_solution_secondary difmap

_refine_ls_shift/su_max 0.000

_refine_ls_shift/su_mean 0.000

_computing_data_collection 'APEX2 (Bruker, 2007)'

_computing_cell_refinement 'SAINT (Bruker, 2007)'

_computing_data_reduction 'SAINT (Bruker, 2007)'

_computing_structure_solution 'SHELXT-2015 (Sheldrick, 2015)'

_computing_structure_refinement 'SHELXL-2019/2 (Sheldrick, 2019)'

_computing_molecular_graphics

'ORTEP-3 for Windows (Farrugia, 2012) and PLATON (Spek, 2009)'

_computing_publication_material

;

WinGX (Farrugia, 2012) and PLATON (Spek, 2009)

;

loop_

_atom_site_label

_atom_site_type_symbol

_atom_site_fract_x

_atom_site_fract_y

_atom_site_fract_z

_atom_site_U_iso_or_equiv

_atom_site_adp_type

_atom_site_occupancy

_atom_site_site_symmetry_order

_atom_site_calc_flag

_atom_site_refinement_flags_posn

_atom_site_refinement_flags_adp

_atom_site_refinement_flags_occupancy

_atom_site_disorder_assembly

_atom_site_disorder_group

O1 O 0.10409(10) 0.1072(2) 0.16956(6) 0.0408(4) Uani 1 1 d . . . . .

O2 O 0.14532(11) 0.4216(3) 0.21617(6) 0.0489(5) Uani 1 1 d . . . . .

O3 O 0.02606(10) -0.2416(3) 0.03233(6) 0.0428(4) Uani 1 1 d . . . . .

C1 C 0.12653(14) 0.2649(3) 0.09683(9) 0.0361(5) Uani 1 1 d . . . . .

C2 C 0.12498(14) 0.2710(3) 0.14481(9) 0.0356(5) Uani 1 1 d . . . . .

C3 C 0.14809(14) 0.4352(4) 0.16897(9) 0.0388(6) Uani 1 1 d . . . . .

C4 C 0.17201(14) 0.5974(4) 0.14479(10) 0.0431(6) Uani 1 1 d . . . . .

H4 H 0.1873 0.7104 0.1609 0.052 Uiso 1 1 calc R U . . .

C5 C 0.17355(14) 0.5940(3) 0.09711(10) 0.0429(6) Uani 1 1 d . . . . .

H5 H 0.1902 0.7052 0.0807 0.051 Uiso 1 1 calc R U . . .

C6 C 0.15136(14) 0.4322(3) 0.07323(10) 0.0415(6) Uani 1 1 d . . . . .

H6 H 0.1527 0.4328 0.0405 0.050 Uiso 1 1 calc R U . . .

C7 C 0.02382(16) 0.1177(4) 0.19020(9) 0.0463(6) Uani 1 1 d . . . . .

H7A H 0.0171 0.0122 0.2122 0.069 Uiso 1 1 calc R U . . .

H7B H -0.0186 0.1080 0.1662 0.069 Uiso 1 1 calc R U . . .

H7C H 0.0179 0.2401 0.2064 0.069 Uiso 1 1 calc R U . . .

C8 C 0.16035(18) 0.5934(4) 0.24129(10) 0.0568(8) Uani 1 1 d . . . . .

H8A H 0.1538 0.5687 0.2744 0.085 Uiso 1 1 calc R U . . .

H8B H 0.1208 0.6919 0.2316 0.085 Uiso 1 1 calc R U . . .

H8C H 0.2168 0.6379 0.2352 0.085 Uiso 1 1 calc R U . . .

C9 C 0.10013(14) 0.0913(3) 0.07268(9) 0.0370(6) Uani 1 1 d . . . . .

H9 H 0.0696 0.0026 0.0908 0.044 Uiso 1 1 calc R U . . .

C10 C 0.11260(14) 0.0394(3) 0.02889(8) 0.0353(5) Uani 1 1 d . . . . .

C11 C 0.07701(13) -0.1422(3) 0.01166(8) 0.0356(6) Uani 1 1 d . . . . .

C12 C 0.11160(13) -0.1831(3) -0.03459(8) 0.0349(5) Uani 1 1 d . . . . .

C13 C 0.16969(14) -0.0223(3) -0.04778(8) 0.0381(6) Uani 1 1 d . . . . .

H13A H 0.1538 0.0325 -0.0780 0.046 Uiso 1 1 calc R U . . .

H13B H 0.2273 -0.0699 -0.0498 0.046 Uiso 1 1 calc R U . . .

C14 C 0.16204(15) 0.1303(3) -0.00939(8) 0.0398(6) Uani 1 1 d . . . . .

H14A H 0.2175 0.1682 0.0019 0.048 Uiso 1 1 calc R U . . .

H14B H 0.1334 0.2457 -0.0212 0.048 Uiso 1 1 calc R U . . .

C15 C 0.08736(14) -0.3402(3) -0.05773(8) 0.0368(6) Uani 1 1 d . . . . .

H15 H 0.0499 -0.4203 -0.0414 0.044 Uiso 1 1 calc R U . . .

C16 C 0.10911(14) -0.4078(3) -0.10365(8) 0.0344(5) Uani 1 1 d . . . . .

C17 C 0.06035(15) -0.5514(3) -0.12342(9) 0.0408(6) Uani 1 1 d . . . . .

H17 H 0.0161 -0.6052 -0.1062 0.049 Uiso 1 1 calc R U . . .

C18 C 0.07545(16) -0.6163(4) -0.16752(10) 0.0467(7) Uani 1 1 d . . . . .

H18 H 0.0404 -0.7112 -0.1806 0.056 Uiso 1 1 calc R U . . .

C19 C 0.14073(16) -0.5452(4) -0.19294(10) 0.0450(6) Uani 1 1 d . . . . .

H19 H 0.1509 -0.5906 -0.2233 0.054 Uiso 1 1 calc R U . . .

C20 C 0.19114(15) -0.4067(4) -0.17346(9) 0.0425(6) Uani 1 1 d . . . . .

H20 H 0.2369 -0.3586 -0.1905 0.051 Uiso 1 1 calc R U . . .

C21 C 0.17610(14) -0.3380(3) -0.12996(9) 0.0380(6) Uani 1 1 d . . . . .

H21 H 0.2112 -0.2422 -0.1174 0.046 Uiso 1 1 calc R U . . .

loop_

_atom_site_aniso_label

_atom_site_aniso_U_11

_atom_site_aniso_U_22

_atom_site_aniso_U_33

_atom_site_aniso_U_23

_atom_site_aniso_U_13

_atom_site_aniso_U_12

O1 0.0441(10) 0.0286(8) 0.0499(10) 0.0120(8) 0.0011(8) 0.0024(7)

O2 0.0506(11) 0.0414(10) 0.0547(11) 0.0003(9) -0.0110(9) -0.0037(9)

O3 0.0387(9) 0.0449(10) 0.0448(10) 0.0129(8) 0.0016(8) -0.0147(8)

C1 0.0275(11) 0.0285(12) 0.0524(15) 0.0086(11) 0.0000(11) -0.0001(10)

C2 0.0263(11) 0.0276(11) 0.0531(15) 0.0116(11) -0.0018(11) 0.0018(10)

C3 0.0271(12) 0.0376(13) 0.0517(15) 0.0058(12) -0.0067(11) 0.0014(10)

C4 0.0283(12) 0.0314(13) 0.0696(19) 0.0029(13) -0.0059(12) -0.0051(10)

C5 0.0309(13) 0.0316(13) 0.0661(18) 0.0145(12) 0.0000(12) -0.0054(10)

C6 0.0339(13) 0.0339(13) 0.0568(16) 0.0141(12) 0.0016(12) -0.0019(11)

C7 0.0460(15) 0.0371(13) 0.0558(16) 0.0128(12) 0.0004(13) -0.0058(12)

C8 0.0531(17) 0.0534(17) 0.0640(19) -0.0073(15) -0.0195(15) -0.0058(14)

C9 0.0301(12) 0.0305(12) 0.0503(15) 0.0146(11) -0.0003(11) -0.0010(10)

C10 0.0290(12) 0.0334(12) 0.0435(14) 0.0154(11) -0.0021(10) -0.0020(10)

C11 0.0259(11) 0.0374(13) 0.0436(14) 0.0173(11) -0.0071(10) -0.0034(10)

C12 0.0265(11) 0.0370(13) 0.0413(13) 0.0180(11) -0.0047(10) -0.0038(10)

C13 0.0330(12) 0.0387(13) 0.0426(14) 0.0181(11) -0.0029(11) -0.0054(11)

C14 0.0355(13) 0.0358(13) 0.0482(15) 0.0152(11) -0.0017(11) -0.0062(11)

C15 0.0283(11) 0.0384(13) 0.0438(14) 0.0187(11) -0.0026(10) -0.0043(10)

C16 0.0292(11) 0.0328(12) 0.0413(13) 0.0155(10) -0.0036(10) -0.0001(10)

C17 0.0335(12) 0.0350(13) 0.0539(16) 0.0111(12) 0.0028(12) -0.0053(11)

C18 0.0406(14) 0.0383(14) 0.0611(18) 0.0006(13) 0.0026(13) -0.0021(11)

C19 0.0423(14) 0.0345(13) 0.0583(17) 0.0019(12) 0.0058(13) 0.0078(11)

C20 0.0354(13) 0.0370(13) 0.0551(16) 0.0106(12) 0.0078(12) 0.0036(11)

C21 0.0315(12) 0.0327(12) 0.0497(15) 0.0111(11) -0.0029(11) -0.0020(10)

_geom_special_details

;

All esds (except the esd in the dihedral angle between two l.s. planes)

are estimated using the full covariance matrix. The cell esds are taken

into account individually in the estimation of esds in distances, angles

and torsion angles; correlations between esds in cell parameters are only

used when they are defined by crystal symmetry. An approximate (isotropic)

treatment of cell esds is used for estimating esds involving l.s. planes.

;

loop_

_geom_bond_atom_site_label_1

_geom_bond_atom_site_label_2

_geom_bond_distance

_geom_bond_site_symmetry_2

_geom_bond_publ_flag

O1 C2 1.391(3) . ?

O1 C7 1.430(3) . ?

O2 C3 1.373(3) . ?

O2 C8 1.424(3) . ?

O3 C11 1.232(3) . ?

C1 C2 1.392(3) . ?

C1 C6 1.412(3) . ?

C1 C9 1.463(3) . ?

C2 C3 1.395(3) . ?

C3 C4 1.386(3) . ?

C4 C5 1.383(4) . ?

C4 H4 0.9500 . ?

C5 C6 1.373(4) . ?

C5 H5 0.9500 . ?

C6 H6 0.9500 . ?

C7 H7A 0.9800 . ?

C7 H7B 0.9800 . ?

C7 H7C 0.9800 . ?

C8 H8A 0.9800 . ?

C8 H8B 0.9800 . ?

C8 H8C 0.9800 . ?

C9 C10 1.336(3) . ?

C9 H9 0.9500 . ?

C10 C11 1.479(3) . ?

C10 C14 1.508(3) . ?

C11 C12 1.481(3) . ?

C12 C15 1.344(3) . ?

C12 C13 1.512(3) . ?

C13 C14 1.546(4) . ?

C13 H13A 0.9900 . ?

C13 H13B 0.9900 . ?

C14 H14A 0.9900 . ?

C14 H14B 0.9900 . ?

C15 C16 1.456(3) . ?

C15 H15 0.9500 . ?

C16 C17 1.398(3) . ?

C16 C21 1.411(3) . ?

C17 C18 1.378(4) . ?

C17 H17 0.9500 . ?

C18 C19 1.379(4) . ?

C18 H18 0.9500 . ?

C19 C20 1.385(4) . ?

C19 H19 0.9500 . ?

C20 C21 1.371(4) . ?

C20 H20 0.9500 . ?

C21 H21 0.9500 . ?

loop_

_geom_angle_atom_site_label_1

_geom_angle_atom_site_label_2

_geom_angle_atom_site_label_3

_geom_angle

_geom_angle_site_symmetry_1

_geom_angle_site_symmetry_3

_geom_angle_publ_flag

C2 O1 C7 113.19(18) . . ?

C3 O2 C8 116.5(2) . . ?

C2 C1 C6 117.7(2) . . ?

C2 C1 C9 119.9(2) . . ?

C6 C1 C9 122.4(2) . . ?

O1 C2 C1 119.7(2) . . ?

O1 C2 C3 118.8(2) . . ?

C1 C2 C3 121.5(2) . . ?

O2 C3 C4 124.8(2) . . ?

O2 C3 C2 115.8(2) . . ?

C4 C3 C2 119.5(2) . . ?

C5 C4 C3 119.8(2) . . ?

C5 C4 H4 120.1 . . ?

C3 C4 H4 120.1 . . ?

C6 C5 C4 120.9(2) . . ?

C6 C5 H5 119.6 . . ?

C4 C5 H5 119.6 . . ?

C5 C6 C1 120.7(2) . . ?

C5 C6 H6 119.6 . . ?

C1 C6 H6 119.6 . . ?

O1 C7 H7A 109.5 . . ?

O1 C7 H7B 109.5 . . ?

H7A C7 H7B 109.5 . . ?

O1 C7 H7C 109.5 . . ?

H7A C7 H7C 109.5 . . ?

H7B C7 H7C 109.5 . . ?

O2 C8 H8A 109.5 . . ?

O2 C8 H8B 109.5 . . ?

H8A C8 H8B 109.5 . . ?

O2 C8 H8C 109.5 . . ?

H8A C8 H8C 109.5 . . ?

H8B C8 H8C 109.5 . . ?

C10 C9 C1 129.5(2) . . ?

C10 C9 H9 115.3 . . ?

C1 C9 H9 115.3 . . ?

C9 C10 C11 119.7(2) . . ?

C9 C10 C14 131.7(2) . . ?

C11 C10 C14 108.5(2) . . ?

O3 C11 C10 125.3(2) . . ?

O3 C11 C12 125.7(2) . . ?

C10 C11 C12 108.95(19) . . ?

C15 C12 C11 120.0(2) . . ?

C15 C12 C13 131.3(2) . . ?

C11 C12 C13 108.7(2) . . ?

C12 C13 C14 106.26(19) . . ?

C12 C13 H13A 110.5 . . ?

C14 C13 H13A 110.5 . . ?

C12 C13 H13B 110.5 . . ?

C14 C13 H13B 110.5 . . ?

H13A C13 H13B 108.7 . . ?

C10 C14 C13 106.41(19) . . ?

C10 C14 H14A 110.4 . . ?

C13 C14 H14A 110.4 . . ?

C10 C14 H14B 110.4 . . ?

C13 C14 H14B 110.4 . . ?

H14A C14 H14B 108.6 . . ?

C12 C15 C16 130.6(2) . . ?

C12 C15 H15 114.7 . . ?

C16 C15 H15 114.7 . . ?

C17 C16 C21 117.3(2) . . ?

C17 C16 C15 118.1(2) . . ?

C21 C16 C15 124.6(2) . . ?

C18 C17 C16 121.1(2) . . ?

C18 C17 H17 119.5 . . ?

C16 C17 H17 119.5 . . ?

C17 C18 C19 120.9(3) . . ?

C17 C18 H18 119.6 . . ?

C19 C18 H18 119.6 . . ?

C18 C19 C20 118.9(3) . . ?

C18 C19 H19 120.6 . . ?

C20 C19 H19 120.6 . . ?

C21 C20 C19 121.0(2) . . ?

C21 C20 H20 119.5 . . ?

C19 C20 H20 119.5 . . ?

C20 C21 C16 120.8(2) . . ?

C20 C21 H21 119.6 . . ?

C16 C21 H21 119.6 . . ?

loop_

_geom_torsion_atom_site_label_1

_geom_torsion_atom_site_label_2

_geom_torsion_atom_site_label_3

_geom_torsion_atom_site_label_4

_geom_torsion

_geom_torsion_site_symmetry_1

_geom_torsion_site_symmetry_2

_geom_torsion_site_symmetry_3

_geom_torsion_site_symmetry_4

_geom_torsion_publ_flag

C7 O1 C2 C1 -107.6(2) . . . . ?

C7 O1 C2 C3 75.7(3) . . . . ?

C6 C1 C2 O1 -177.5(2) . . . . ?

C9 C1 C2 O1 4.7(3) . . . . ?

C6 C1 C2 C3 -0.8(3) . . . . ?

C9 C1 C2 C3 -178.7(2) . . . . ?

C8 O2 C3 C4 6.5(3) . . . . ?

C8 O2 C3 C2 -173.7(2) . . . . ?

O1 C2 C3 O2 -2.2(3) . . . . ?

C1 C2 C3 O2 -178.8(2) . . . . ?

O1 C2 C3 C4 177.6(2) . . . . ?

C1 C2 C3 C4 1.0(4) . . . . ?

O2 C3 C4 C5 179.1(2) . . . . ?

C2 C3 C4 C5 -0.7(4) . . . . ?

C3 C4 C5 C6 0.3(4) . . . . ?

C4 C5 C6 C1 -0.1(4) . . . . ?

C2 C1 C6 C5 0.4(3) . . . . ?

C9 C1 C6 C5 178.2(2) . . . . ?

C2 C1 C9 C10 -164.3(2) . . . . ?

C6 C1 C9 C10 18.0(4) . . . . ?

C1 C9 C10 C11 -177.9(2) . . . . ?

C1 C9 C10 C14 5.1(4) . . . . ?

C9 C10 C11 O3 10.7(3) . . . . ?

C14 C10 C11 O3 -171.6(2) . . . . ?

C9 C10 C11 C12 -170.1(2) . . . . ?

C14 C10 C11 C12 7.6(2) . . . . ?

O3 C11 C12 C15 0.2(3) . . . . ?

C10 C11 C12 C15 -179.0(2) . . . . ?

O3 C11 C12 C13 178.2(2) . . . . ?

C10 C11 C12 C13 -1.0(2) . . . . ?

C15 C12 C13 C14 171.9(2) . . . . ?

C11 C12 C13 C14 -5.8(2) . . . . ?

C9 C10 C14 C13 166.3(2) . . . . ?

C11 C10 C14 C13 -11.0(2) . . . . ?

C12 C13 C14 C10 10.2(2) . . . . ?

C11 C12 C15 C16 177.3(2) . . . . ?

C13 C12 C15 C16 -0.2(4) . . . . ?

C12 C15 C16 C17 -165.2(2) . . . . ?

C12 C15 C16 C21 14.6(4) . . . . ?

C21 C16 C17 C18 -2.5(3) . . . . ?

C15 C16 C17 C18 177.3(2) . . . . ?

C16 C17 C18 C19 2.0(4) . . . . ?

C17 C18 C19 C20 -0.1(4) . . . . ?

C18 C19 C20 C21 -1.1(4) . . . . ?

C19 C20 C21 C16 0.5(4) . . . . ?

C17 C16 C21 C20 1.3(3) . . . . ?

C15 C16 C21 C20 -178.5(2) . . . . ?

loop_

_geom_hbond_atom_site_label_D

_geom_hbond_atom_site_label_H

_geom_hbond_atom_site_label_A

_geom_hbond_distance_DH

_geom_hbond_distance_HA

_geom_hbond_distance_DA

_geom_hbond_angle_DHA

_geom_hbond_site_symmetry_A

C14 H14B O3 0.99 2.60 3.206(3) 119.9 5

C17 H17 O3 0.95 2.49 3.319(3) 146.0 5_545

_refine_diff_density_max   0.215

_refine_diff_density_min   -0.174

_refine_diff_density_rms   0.042
